# Impact of direct physical association and motility on fitness of a synthetic interkingdom microbial community

**DOI:** 10.1038/s41396-022-01352-2

**Published:** 2022-12-24

**Authors:** Giovanni Scarinci, Victor Sourjik

**Affiliations:** Max Planck Institute for Terrestrial Microbiology and Center for Synthetic Microbiology (SYNMIKRO), Marburg, Germany

**Keywords:** Microbial ecology, Environmental microbiology

## Abstract

Mutualistic exchange of metabolites can play an important role in microbial communities. Under natural environmental conditions, such exchange may be compromised by the dispersal of metabolites and by the presence of non-cooperating microorganisms. Spatial proximity between members during sessile growth on solid surfaces has been shown to promote stabilization of cross-feeding communities against these challenges. Nonetheless, many natural cross-feeding communities are not sessile but rather pelagic and exist in turbulent aquatic environments, where partner proximity is often achieved via direct cell-cell adhesion, and cooperation occurs between physically associated cells. Partner association in aquatic environments could be further enhanced by motility of individual planktonic microorganisms. In this work, we establish a model bipartite cross-feeding community between bacteria and yeast auxotrophs to investigate the impact of direct adhesion between prokaryotic and eukaryotic partners and of bacterial motility in a stirred mutualistic co-culture. We demonstrate that adhesion can provide fitness benefit to the bacterial partner, likely by enabling local metabolite exchange within co-aggregates, and that it counteracts invasion of the community by a non-cooperating cheater strain. In a turbulent environment and at low cell densities, fitness of the bacterial partner and its competitiveness against a non-cooperating strain are further increased by motility that likely facilitates partner encounters and adhesion. These results suggest that, despite their potential fitness costs, direct adhesion between partners and its enhancement by motility may play key roles as stabilization factors for metabolic communities in turbulent aquatic environments.
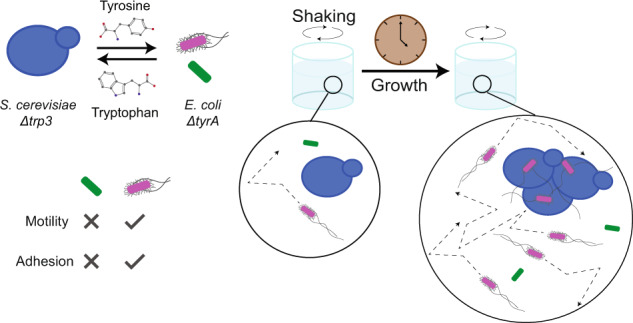

## Introduction

Organisms living in natural microbial consortia often share metabolites with the other community members. One of the possible metabolic interactions is represented by mutualistic cross-feeding [1]. This relies on the reciprocal exchange of essential metabolites between partners whose production of the received compound is lacking, implying a division of labor within the consortium for the synthesis of such traded building blocks. The derived strong inter-organisms dependence has even been proposed to be a crucial step towards the establishment of more complex symbiotic relationships [2]. However, such cross-feeding communities face several challenges that might hinder their growth and stability. One major problem is the rapid dispersal and subsequent loss of the exchanged compounds, as they are released in the environment [3]. Another challenge is the exploitation of shared metabolites by non-cooperating (“cheating”) organisms, which can reduce community growth and even lead to its collapse [4–6]. A common strategy that might be used by mutualistic communities to counteract these negative effects is the spatial assortment of partners [7, 8]. As predicted by theory and confirmed by experimental studies of model synthetic communities grown on an agar surface or in a microfluidic chamber, stability of cross-feeding communities can benefit from positive spatial assortment between partners that facilitates short-range metabolic interactions and prevents exploitation by cheaters [9–14]. Consistently, spatial structuring spontaneously emerges in cross-feeding communities grown on agar surfaces [12, 14–18]. Moreover, structuring might favor the evolution of bacterial cross-feeding [19] and be itself enhanced by such evolution [20]. An extreme example of assortment is direct channeling of metabolites between two cooperating partners, as occurs in endosymbiosis [21–23] but also in cross-feeding bacterial communities [19, 24, 25].

Although partners proximity in natural sessile communities can similarly result from passive spatial assortment during growth on a surface [26], it can be alternatively achieved through direct physical interactions between partners at both intra- and interkingdom levels [27, 28]. Such adhesion may be especially important for the suspended aggregates [29, 30] as well as for epibionts [31–34], communities in which one of the partners, usually smaller, grows on the surface of the other partner. Even for such closely associated communities, it typically remains unknown whether the physical interaction can enhance metabolite exchange on its own, or rather stabilize specific structures involved in direct metabolite transfer [1, 3]. Furthermore, in open aquatic environments where non-surface attached communities are typically found [35], the ability of motile planktonic microorganisms to swim can be beneficial in the search for partners [36–38]. But despite their assumed importance, joint impact of physical association and motility remains little studied because of the inherent complexity of microbial interactions in natural communities [39].

To quantitatively characterize the importance of these factors in a well-defined model community, we established a synthetic interkingdom cross-feeding consortium between the bacterium *Escherichia coli* and the budding yeast *Saccharomyces cerevisiae*, in which the physical association between the two partners is mediated by the bacterial type I fimbriae [40] and both adhesion and bacterial motility could be easily manipulated. We demonstrate that cell adhesion, and possibly the resulting cell clumping, provide moderate but significant competitive advantage to fimbriated bacteria in a well-mixed co-culture with yeast, despite modest reduction of the overall community growth. Physical interaction was further important to exclude non-cooperating bacterial cheaters, thereby delaying the collapse of the community upon cheater invasion. Swimming provides an additional fitness advantage to the adhesive bacterial partner and further increases its competitiveness against the cheater. These findings demonstrate that investments in adhesion and motility can be beneficial for mutualistic microbial communities existing in turbulent aquatic environments.

## Materials and methods

### Strain construction

*E. coli* strains were derived from BW25113 or, for motility studies, MG1655 [41]. Mutant strains were generated via P1 transduction or λ-red recombinase using the pSIJ8 plasmid [42]. *E. coli* strains in the co-culture were labelled either with a sfGFP-expressing plasmid (pTrc99A:sfGFP: pNB1) or with an mCherry-expressing plasmid (pTrc99A:mCherry: pOB2), both inducible by isopropyl-β-D-thiogalactopyranoside (IPTG). For co-cultures grown in minimal media, sufficient expression of fluorescent markers was observed even without IPTG induction, but 10 µM IPTG was used for cultures grown in supplemented media. All *S. cerevisiae* strains were obtained from the gene knockout collection (Dharmacon, Lafayette, CO, USA) [43] derived from the BY4741 strain. The *his3Δ1* auxotrophy of this strain was rescued by an insertion restoring histidine prototrophy and introducing mTurquoise2 as a fluorescent marker. A complete list of the strains and plasmids used in this study can be found in Table S1.

### Growth conditions

For pre-cultures, *S. cerevisiae* cells were streaked from glycerol stocks on yeast extract peptone dextrose (YPD) plates supplemented with the appropriate antibiotic and incubated at 30 °C for 48 h. From each plate, four to six colonies were inoculated in 5 mL YPD supplemented with the appropriate antibiotic, and cells were grown at 30 °C for 16–18 h with shaking at 200 r.p.m. *E. coli* pre-cultures were inoculated directly from glycerol stocks in 5 mL lysogeny broth (LB) with the appropriate antibiotic and grown at 37 °C for 16–18 h with shaking at 200 r.p.m.

For both organisms, cells from 2 mL pre-culture were washed twice with phosphate-buffered saline (PBS), resuspended in 1 mL PBS and incubated for 5 h, at 30 °C degrees for *S. cerevisiae* and 37 °C for *E. coli*. Unless indicated otherwise, cells were diluted to a total OD_600_ of 0.05 for *S. cerevisiae* and a total of 0.025 for *E. coli* partner strain(s), values referring to a 1 cm cuvette. When the *E. coli* cheater was introduced in the community, an initial inoculum of 0.025 (for 50% inoculation) or 0.011 (for 30% inoculation) of this strain was further added to the culture. Growth and competition assays were performed at 30 °C with shaking at 200 r.p.m. in 24-well microtiter plates (Greiner Bio-One, Frickenhausen, Germany) in 1 mL low fluorescence (LoFlo) yeast nitrogen base (YNB) minimal media (Formedium, Swaffham, UK) buffered with 100 mM 2-(*N*-morpholino) ethanesulfonic acid (MES) (Roth, Karlsruhe, Germany) at pH 6.15 and with 2% d-glucose, or 1% d-fructose for the motility assay, as carbon source. For the cross-feeding experiments the media was supplemented with a mixture of 100 mg/L l-leucine, 20 mg/L l-methionine and 20 mg/L uracil to complement the auxotrophies present in the *S. cerevisiae* background strain. For the controls experiments with fully supplemented media the complete supplement mixture (CSM, a mixture of diverse amino acids, Foremedium) enriched with 20 mg/L l-serine was used. Where indicated, 4% d-(+)-mannose was introduced into the media. For the co-cultures grown in a semi-continuous mode, 500 µL of each culture were transferred to a new well containing 500 µL of fresh media every 24 h for 10 days. For all the experiments, growth was measured using a plate reader (m200 Infinite Pro, Tecan, Männedorf, Switzerland). When present, clumps were disrupted prior to measurements or culture transfer by pipetting the sample up and down 10 times with a 1 mL pipette.

Colony assays were performed on minimal media plates containing 1% agarose where 2 µL of cell mixture with the same initial concentration as for liquid cultures were deposited. Plates were incubated at 30 °C for ten days to reach the maximal colony size.

### Flow cytometry

Flow cytometry was performed with BD LSR Fortessa SORP cell analyzer (BD Biosciences, Heidelberg, Germany). GFP fluorescence was detected using a 488 nm laser line combined with a 510/20 BP filter. mTurquoise2 fluorescence was measured using a 447 nm laser line combined with a 470/15BP filter. mCherry fluorescence was measured using a 561 nm laser line combined with 632/22 BP filter. *S. cerevisiae* and *E. coli* populations were distinguished using forward scatter (FSC) and side scatter (SSC). *E. coli* strains were further distinguished according to their respective fluorescent labelling (mCherry or GFP). Before the measurements, cell aggregates were disrupted by pipetting as described above, followed by a dilution in a ratio 1:10 in PBS supplemented with 4% mannose (1 mL final volume). Measurements were performed using the BD High Throughput Sampler (HTS) with a fixed flow rate set at 1 µL/s for an acquisition time of 20 s with samples diluted to a concentration typically of 10^3^–10^4^ events per second in PBS supplemented with 4% mannose. Dilution rates, flow rate and sampling time were then used to infer the abundance of cells in the defined volume (20 µL). Flow cytometry results were analyzed using FlowJo (BD Biosciences). The detailed description of all the statistical analysis performed in this work can be found in the Supplementary Materials and methods section.

### Microscopy imaging

Confocal images acquisitions were performed on a Zeiss LSM-800 microscope, using 594 nm laser line acquiring in the 606–695 nm window for mCherry, 488 nm laser line acquiring in the 535–580 nm window for sGFP, and 440 nm laser line acquiring in the 446–491 nm window for mTurquoise2. For the aggregation assay, samples were first deposited on a 1% agarose pad and covered by a coverslip, and a Plan-Apochromat 63×/1.40 Oil objective was used. For colony imaging, samples were prepared by depositing colonies upside down in 8-well glass-bottom slides (μ-Slide, 8-well glass bottom; ibidi), embedded in 250 µL of 1% low melting agarose (A9414, Sigma, St. Louis, MO, USA) in PBS and incubating at room temperature for 10 min. The acquisition was performed using a LD LCI Plan-Apochromat 25x/0.8 Imm Corr objective. Images from experiments aimed to assess clump disruption were acquired using a Nikon Eclipse Ti-U fluorescence microscope (Nikon Instruments, Japan) with a 20x objective and a Zyla 4.2 Plus sCMOS camera (Andor Technology Ltd, Belfast, UK).

## Results

### Physical interaction with yeast provides selective advantage to bacteria in a cross-feeding community

In order to investigate the effects of physical association on interkingdom metabolic cooperation, we exploited the ability of *E. coli* to bind to surface mannoproteins of *S. cerevisiae* via type I fimbriae [40]. Indeed, we observed association between bacteria and yeast cells and their ensuing co-aggregation in a co-culture (Fig. S1A, Supplementary Materials and methods). These interactions were abolished when mannose was added to the culture medium (Fig. S1B), or in *E. coli* mutants lacking either the mannose-binding fimbrial tip FimH (Fig. S1C) or the major subunit of fimbriae FimA (Fig. S1D). This specific co-aggregation in the co-culture of yeast with the fimbriated *E. coli* could be confirmed by the analysis of microscopy images, where *S. cerevisiae* cells displayed a significantly higher spatial autocorrelation and cross-correlation with *E. coli* cells compared to all controls where binding was prevented (Fig. S1E–I, Supplementary Materials and methods).

**Fig. 1:**
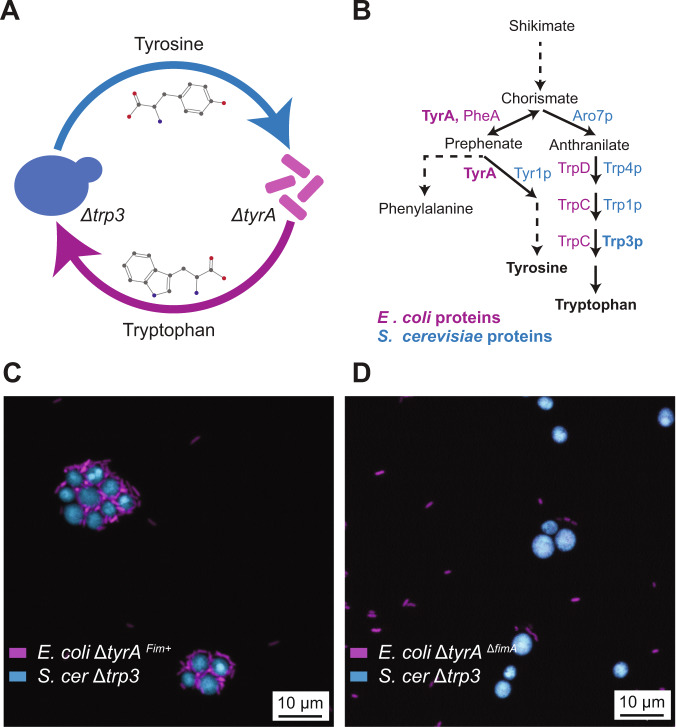
Engineering of physically interacting cross-feeding community. **A** Schematic representation of metabolic dependencies within the engineered community. *E. coli* (magenta) is auxotroph for tyrosine while *S. cerevisiae* (blue) is auxotroph for tryptophan. **B** Biosynthetic pathway for aromatic amino acids. Arrows represent individual reactions, with the corresponding enzymes shown both for *E. coli* (magenta) and for *S. cerevisiae* (blue). Enzymes that correspond to gene knockouts used in this study are indicated in bold. **C**, **D** Confocal microscopy images of the engineered communities with *S. cerevisiae* expressing mTurquoise2 (blue) mixed either with (C) Fim^+^ or (D) Δ*fimA E. coli* expressing mCherry (magenta). Scale bar = 10 µm.

**Fig. 2:**
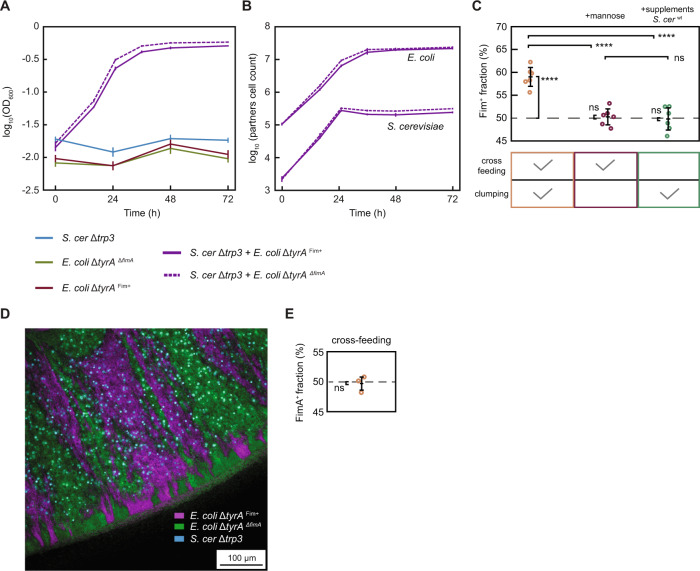
Characterization of the engineered cross-feeding community. **A** Growth of the co-cultures incubated in the selective YNB-glucose minimal medium, measured as optical density at 600 nm. Solid and dashed magenta lines indicate communities containing Fim^+^ or Δ*fimA E. coli*, respectively. Monocultures of each strain are shown as controls. Error bars represent standard deviations of three biological replicates. **B** Numbers of *S. cerevisiae* (labelled with mTurquoise2) and *E. coli* (labelled with mCherry) cells measured by flow cytometry in 20 µL of the same co-cultures as in (**A**). **C** Fraction of Fim^+^ cells (labelled with mCherry) in the total *E. coli* population when co-cultured with Δ*fimA E. coli* (labelled with sfGFP) and *S. cerevisiae* (labelled with mTurquoise2). Co-cultures were inoculated with equal amounts of Fim^+^ and Δ*fimA* cells and grown for 72 h either in YNB-glucose, in YNB-glucose supplemented with 4% mannose (no clumping) or YNB-glucose supplemented with complete supplement mixture (CSM; no cross-feeding), as indicated. In the latter case the yeast auxotroph was additionally replaced by the parental prototroph. Error bars represent standard deviations derived from six biological replicates (indicated by circles). ****p ≤ 0.0001 in either a two-tailed *t*-test assuming equal variances of the data sets or in a one sample *t*-test assessing difference from a 50% fraction average. *ns*, non-significant. Confocal microscopy image of a colony sector (**D**), and the fraction of Fim^+^ cells (**E**), for co-culture of *S. cerevisiae* (blue) and equal amounts of Fim^+^ (magenta) and Δ*fimA* (green) *E. coli* that was grown on a 1% agarose plate for 10 days. Scale bar in (**D**) = 100 µm. Cells were labelled as in (**C**). *ns*, not significant in a one sample *t*-test assessing difference from a 50% fraction average.

To introduce mutualistic cross-feeding, this fimbriae-mediated physical association was combined with an obligate metabolic dependency between *E. coli* Δ*tyrA* strain that requires tyrosine for growth and *S. cerevisiae* Δ*trp3* strain requiring tryptophan (Fig. 1A, B; Fig. S2A, B). This cross-feeding community showed fimbriae-dependent co-aggregation between bacteria and yeast and robust growth in co-culture (Fig. 1C, D, Fig. 2A and Fig. S3A–C). Despite their metabolic interdependence, the two organisms showed differences in their time course of growth, with *S. cerevisiae* reaching the maximal cell density earlier than *E. coli* (Fig. 2B). Although the expression of fimbriae in *E. coli* is known to be phase-variable [44], we confirmed that the fraction of fimbria-expressing cells was very high (~90%) under our conditions, implying that the majority of genetically Fim^+^ *E. coli* cells possess the ability to adhere to yeast (Fig. S5, Supplementary Materials and methods).

While comparable growth profiles were observed when this synthetic community was assembled with either Fim^+^ or fimbrialess (Δ*fimA*) *E. coli*, both the overall final density of the co-culture and the numbers of *S. cerevisiae* and *E. coli* cells were slightly but significantly reduced when *E. coli* was fimbriated (Fig. 2B; Fig. S4A-C). This reduction could be due to the formation of multicellular clumps in the community containing fimbriated *E. coli* (Fig. 1C). The impact of aggregation was indeed also observed in the minimal medium supplemented with a mixture of amino acids (Fig. S6A). In this case, only the number of *S. cerevisiae* cells was reduced, which was mirrored by the increased number of *E. coli* cells (Fig. S6B). These results indicate that co-aggregation directly affects yeast growth, and indirectly reduces *E. coli* growth in a cross-feeding community but not in the supplemented medium where the two organisms rather exhibit competitive behavior.

Physical association might nevertheless confer a competitive advantage to fimbriated *E. coli* cells in the cross-feeding community, by ensuring their stable association and efficient intermixing with the yeast partner and an enhanced access to exchanged metabolites within co-aggregates (Fig. 1C). In order to test this directly, we co-cultured fimbriated and fimbrialess *E. coli*, each labelled with a different fluorescent marker, with the yeast auxotroph. The abundances of each community member were assessed by flow cytometry after complete disruption of cell aggregates (Fig. S7). Although in these experiments both *E. coli* strains were inoculated in equal amounts, we observed that fimbriated cells were significantly more abundant in the final community, and this enrichment was dependent on the interaction with the yeast partner and on cross-feeding (Fig. 2C) and was not influenced by the choice of reporter (Fig. S8). This increase in abundance was even more pronounced at lower inoculation density (Fig. S9), likely due to enhanced competition for exchanged metabolites when the cross-feeding partners are scarce. In contrast to previous observations in bacteria [19, 24, 25, 45], the beneficial effect of adhesion in our synthetic interkingdom community appears to rely on exchange of diffusible metabolites rather than on direct cytoplasmic connections between partners (Fig. S10). A comparable enrichment of fimbriated *E. coli* was observed in a cross-feeding co-culture of *E. coli* Δ*tyrA* with Δ*trp4* strain of *S. cerevisiae* that is interrupted at a different step in the tryptophan biosynthetic pathway (Fig. 1B; Fig. S11).

In contrast to the co-culture grown in liquid media, there was no significant increase in abundance of fimbriated *E. coli* cells in communities grown on the surface of a minimal media agar plate, indicating that fimbriation provides no competitive advantage under these conditions (Fig. 2D, E). This difference from the well-mixed liquid culture might be due to the spatial segregation between fimbriated and fimbrialess *E. coli* strains into different sectors of the colony, as observed previously [10, 14, 16, 46], which prevents their local competition.

### Partner adhesion reduces invasion of community by a cheater

**Fig. 3:**
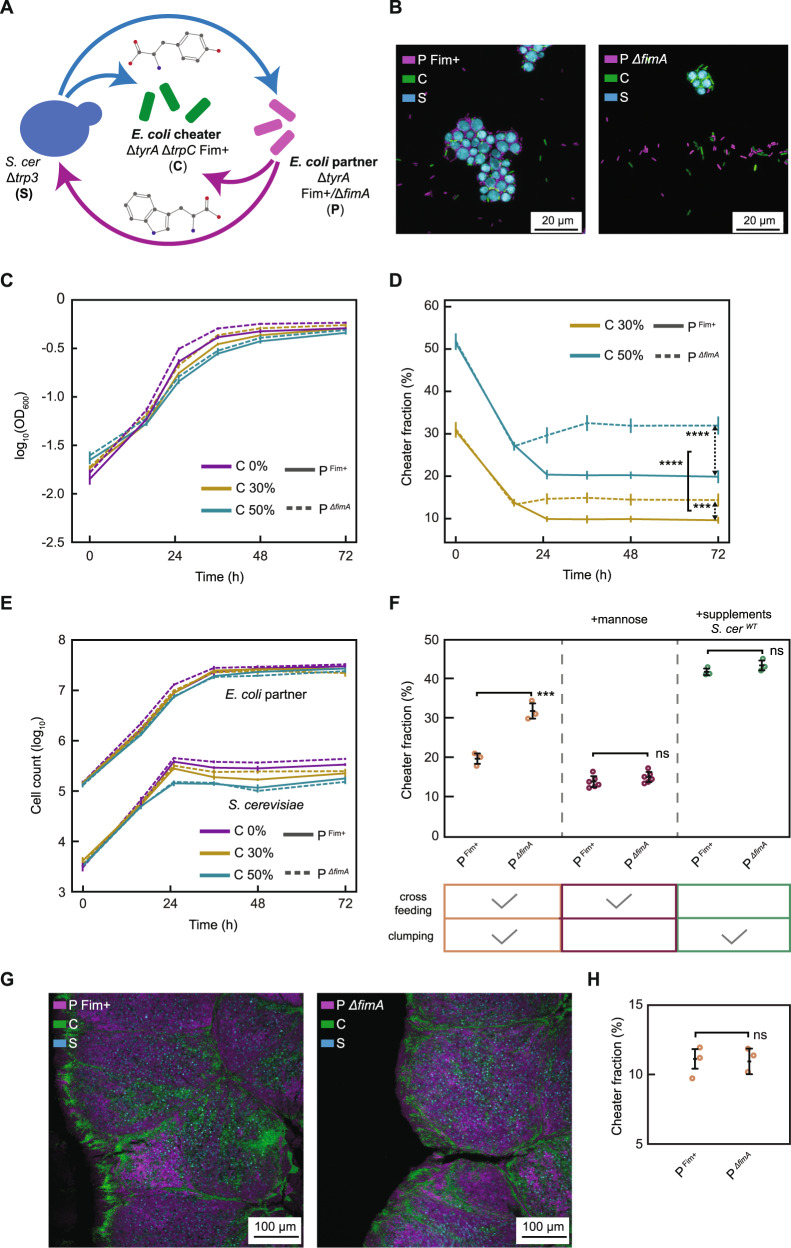
Partner adhesion reduces abundance of a non-cooperating cheater. **A** Schematic representation of metabolic dependencies within the engineered community containing a non-cooperating *E. coli* cheater (labeled “C”; green), that is auxotroph for both tyrosine and tryptophan in co-culture with *S. cerevisiae* Δ*trp3* (labeled “S”; turquoise), and either a fimbriated (Fim^+^) or a fimbrialess (Δ*fimA*) *E. coli* partner (labeled “P”; magenta). **B** Confocal microscopy images of cell clusters of *S. cerevisiae* expressing mTurquoise2 (blue) and either Fim^+^ or Δ*fimA E. coli* partners expressing mCherry (magenta), in the presence of an *E. coli* Fim^+^ cheater expressing sfGFP (green). Scale bar = 20 µm. **C** Growth of the co-cultures in the selective YNB-glucose minimal medium in the presence of 50% (turquoise) or 30% (gold) cheater. Solid and dashed lines indicate communities containing Fim^+^ or Δ*fimA E. coli* partner, respectively. Error bars represent standard deviations of three biological replicates. **D** Fraction of cheater in the total *E. coli* population in the same co-cultures as in (**C**). ***p < 0.001 and ****p < 0.0001 from one tailed *t*-test assuming equal variances of the data sets, with large effect size (Cohen’s *d* > 1) for each comparison. **E** Numbers of *S. cerevisiae* (labelled with mTurquoise2) and *E. coli* partner (labelled with mCherry) cells measured by flow cytometry in 20 µL of the same co-cultures as in (**C**). ***p < 0.001 from a post hoc analysis (Tukey HSD) performed after a two-way ANOVA confirming interaction between growth condition and the effect of fimbriation (Table S2). **F** Fraction of cheater in communities containing either Fim^+^ or Δ*fimA E. coli* partner at the initial 50% abundance of cheater, grown in YNB-glucose (orange) (Data from **C**), YNB-glucose supplemented with 4% mannose (red) and in in YNB-glucose supplemented with CSM and with *S. cerevisiae* prototroph (green). ****p < 0.0001 from two tailed *t*-test assuming equal variances of the data sets for three to six biological replicates, with very large effect size (Cohen’s *d* > 5). *ns*, non-significant, *p* > 0.15. Confocal microscopy image of a colony sector (**G**) and the fraction of cheater in the total *E. coli* population (**H**) from co-cultures of *S. cerevisiae* with either Fim^+^ or Δ*fimA E. coli* as indicated, and the cheater at the initial 50% abundance, grown on a 1% agarose plate for 10 days. *ns*, non-significant, from two tailed *t*-test assuming equal variances of the data sets for three biological replicates.

One potential ecological benefit of the physical association between species in communities could reside in protection against the exploitation of shared metabolites by non-cooperators that do not contribute to the consortium. We thus engineered an *E. coli* strain with disruptions in both the tryptophan and tyrosine biosynthetic pathways (Δ*tyrA* Δ*trpC*) that requires supplementation of both amino acids for growth (Fig. 3A; Fig. S12). This strain behaves as a non-cooperating cheater exploiting metabolites released by both partners for its own growth without providing any benefits to the community, and it mimics a plausible scenario how natural cheaters could emerge by gene loss [47–49]. When this fimbriated cheater strain was introduced in our community along with the partner (Δ*tyrA*) *E. coli* strain, it showed localization to bacteria-yeast aggregates (Fig. 3B) and growth within community but not with individual partners (Figs. S12 and S13). The introduction of the cheater led to a small but significant reduction of the community growth rate and of the final OD, dependent on the dose of the cheater (Fig. 3C, D; Fig. S14A, B, D, E). The number of yeast cells in the community was also significantly reduced in the presence of the cheater (Fig. 3E; Fig. S14C, F). The community containing the fimbrialess *E. coli* was more strongly affected by the initial dose of the cheater, i.e., showed higher slopes of the regression fits in Fig. S14A–I. This was apparently due to the reduced final cheater abundance in the presence of partner fimbriation (Fig. 3D), since the regression lines became similar for both communities when plotted against the final cheater abundance, apart from differences due to the direct reduction of community growth caused by *E. coli* fimbriation (Fig. S14G–I). Thus, physical association with yeast helps the bacterial partner to outcompete the cheater, and this beneficial effect of association could compensate or, in the presence of higher number of cheater cells, even outweigh the aggregation-dependent reduction of *S. cerevisiae* growth (Fig. S14C, F).

The dependence of cheater fraction and of the total *E. coli* cell count on partner fimbriation was no longer significant in the presence of mannose or in the absence of cross-feeding (Fig. 3F; Table S2). The final fraction of cheater cells in *E. coli* population in the supplemented medium was below 50% (around 40%), indicating moderately lower fitness of the cheater compared to the partner strain in the absence of cross-feeding, most likely due to imperfect compensation of *trpC* deletion effects by supplementing medium with tryptophan. Nevertheless, this cheater fraction was further significantly reduced in the cross-feeding community, to 30% in the presence of the fimbrialess *E. coli* partner and to 20% in the presence of the fimbriated *E. coli* partner, possibly due to the negative selection on cheater-enriched cell aggregates (see “Discussion”). Similarly, beneficial impacts of partner fimbriation were observed when using a fimbrialess cheater strain (Fig. S15) as well as for the alternative yeast tryptophan auxotroph Δ*trp4* (Fig. S16).

The enhancement of partner competitiveness due to its fimbriation was again no longer detectable once the communities containing the cheater were grown on a solid agar surface (Fig. 3G, H). The fraction of the cheater cells in a colony, measured by flow cytometry after colony resuspension, was ~11% and thus generally lower than in the batch culture with or without partner fimbriation (two-tailed *t*-test assuming equal variances of the data sets, *p* < 0.0001). Such lower fitness of cheater might be the consequence of spatial segregation between partner and cheater bacteria within the colony (Fig. 3H; Fig. S17). However, *S. cerevisiae* does not display an apparent segregation from *E. coli* cheaters, most likely because the relatively small size of cheater sectors is below the range of metabolic interactions within the colony [14] (Fig. S17).

**Fig. 4:**
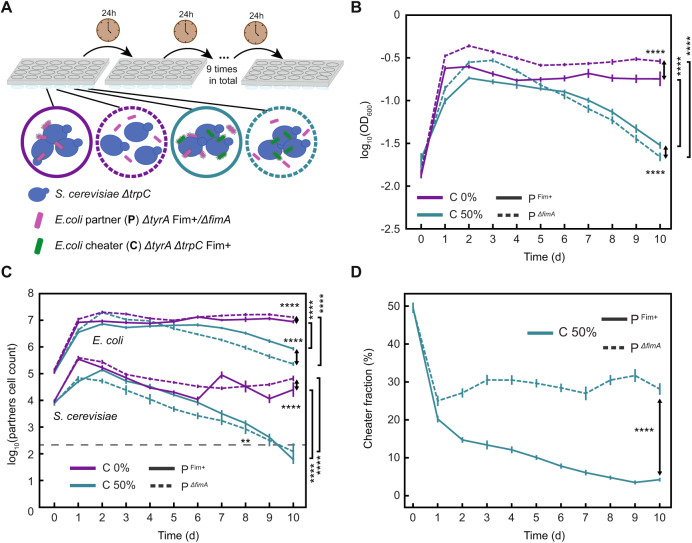
Impact of cheater on communities in a semi-continuous growth mode. **A** Schematic representation of the experimental setup for the semi-continuous growth mode, where 500 µL of the co-cultures are repeatedly transferred to 500 µL fresh media every 24 h for a total of 10 days. **B** Growth of the co-cultures in the selective YNB-glucose minimal medium either in absence (magenta) or in presence (turquoise) of 50% cheater (labeled “C”), as indicated. Solid and dashed lines indicate communities containing either Fim^+^ or Δ*fimA E. coli* partner (labeled “P”), respectively. Error bars represent standard deviations of six to twelve biological replicates. ****p < 0.0001 from one tailed *t*-test assuming equal variances of the data sets, with large effect size (Cohen’s *d* > 1). **C** Numbers of *S. cerevisiae* (labelled with mTurquoise2) and *E. coli* partner (labelled with mCherry) cells measured by flow cytometry in 20 µL of the same co-cultures as in (**B**). For *S. cerevisiae*, the last two time points were excluded since the value obtained is below, or just above the blank control (gray dashed line) ****p < 0.0001 and *0.05 > *p* > 0.01 from one tailed *t*-test assuming equal variances of the data sets, with large effect size (Cohen’s *d* > 1) for each comparison. **D** Fraction of cheater in the cheater-containing communities in (**B**). ****p < 0.0001 from one tailed *t*-test assuming equal variances of the data sets, with large effect size (Cohen’s *d* > 1).

Beyond a single growth cycle of the community, we studied the longer-term impact of partner fimbriation by culturing our community in a semi-continuous growth mode. This was done by transferring an inoculum from the culture to fresh media every 24 h (Fig. 4A). In the absence of the cheater, such repeated transfers eventually resulted in the establishment of a relatively stable community (Fig. 4B, C). Consistently with previous experiments, cell density and *S. cerevisiae* and *E. coli* partner cell counts were lower when the *E. coli* partner was fimbriated (Fig. 4B, C). In contrast, in the presence of the cheater, the community was no longer stable and experienced a gradual decline after an initial phase of increased density, with the count of yeast cells eventually dropping to a number of events comparable to that in the blank, even though the relative abundance of the cheater was low (Fig. 4D). In that case, partner fimbriation became again beneficial, resulting not only in largely reduced cheater and increased *E. coli* partner abundance, but also leading to a significantly higher number of yeast cells over most of the experimental time course (*p* < 0.05 and effect size, calculated as Cohen’s *d*, larger than 1.5 from day 2 until day 8) (Fig. 4C, D; Fig. S18), and consequently significantly delaying community collapse (Fig. 4B). Thus, also under these conditions, the benefit due to the exclusion of the cheater overweighed the immediate negative impact of fimbriation on yeast growth, although it could not prevent the eventual collapse of the community.

### Bacterial motility provides fitness benefit in presence of adhesion

**Fig. 5:**
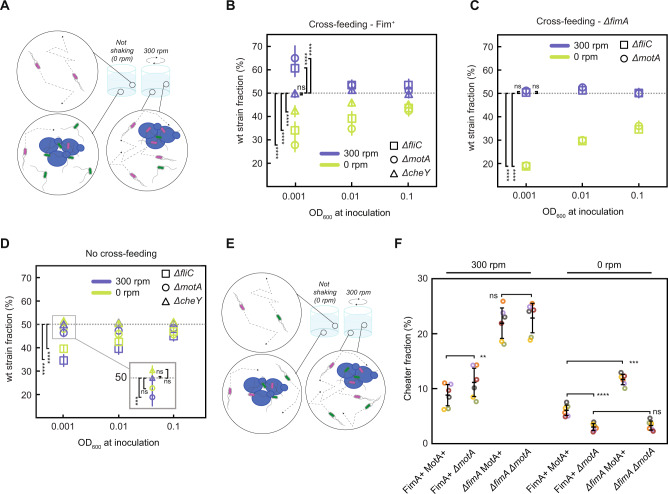
Effects of motility on the bacterial partner fitness in physically interacting community. **A** Schematic representation of the experimental setup to study effects of motility on the community, with yeast-bacteria co-cultures grown either without shaking or with shaking at 300 r.p.m. While without shaking non-motile *E. coli* (e.g. Δ*motA*, green) and yeast cells (blue) sediment to the bottom of the well, and the motile *E. coli* (red) is in suspension, under shaking the entire community is maintained in suspension. **B** Fraction of motile and chemotactic *E. coli* cells (labeled with mCherry) compared to the total *E. coli* population in co-culture with sfGFP-labeled non-motile (either Δ*fliC*, or Δ*motA*) or non-chemotactic (Δ*cheY*) *E. coli* cells and with yeast. Communities were inoculated with different initial optical density (OD) as indicated and grown for 96 h under shaking (blue) or without shaking (light green) in YNB-fructose minimal medium. Error bars represent standard deviations of at least six biological replicates. ****p < 0.0001 and ***p < 0.001 from one sample *t*-test assessing for difference to a 50% fraction average, with large effect size (Cohen’s *d* > 1) for each statistically different comparison. *ns*, non-significant. **C** Fraction of motile and chemotactic cells in the total *E. coli* population in the co-cultures grown like in (**B**) but in Δ*fimA* background. Error bars represent standard deviations of at least six biological replicates. ****p < 0.0001 and ***p < 0.001 from one sample *t*-test assessing for difference to a 50% fraction average, with large effect size (Cohen’s *d* > 1) for each statistically different comparison. *ns*, non-significant. **D** Fraction of motile and chemotactic cells in the total *E. coli* population in co-cultures grown like in (**B**) but supplemented with CSM. **E** Schematic representation of the experimental setup to study effects of motility on the community in presence of a cheater. **F** Cheater fraction from communities with a motile and fimbriated *E. coli* cheater (labeled with sfGFP) compared to the total *E. coli* population co-cultured with yeast and with an mCherry-labeled *E. coli* partner displaying different status of fimbriation and motility, as indicated. Communities were inoculated with an initial 50% cheater fraction and an initial optical density of 0.001 and grown for 96 h under shaking (300 r.p.m) or without shaking (0 r.p.m) in YNB-fructose minimal medium. Error bars represent standard deviations of six biological replicates represented as circles. ****p < 0.0001, ***p < 0.001, **p < 0.01 from paired *t*-test.

In aquatic environments, the ability of cells to actively move could increase the encounter rate between partners, but it might also enhance cell detachment. To test the impact of motility on the relative fitness of bacteria in our community, we performed competition assays between motile and non-motile *E. coli* partners co-cultured with yeast (Fig. 5A). Two different non-motile strains of *E. coli* were used, either deleted for flagellin gene *fliC* and thus lacking flagellar filaments, or deleted for the flagellar motor gene *motA* and displaying non-functional, yet structurally intact, flagella. Moreover, we further tested a motile but non-chemotactic *E. coli* strain (*ΔcheY*) that is no longer capable of following chemical gradients in the environment. Since the motility of the parental strain *E. coli* BW25113 used in the previous experiments is generally poor, and large spontaneous variability of swimming abilities was reported for its derivatives [50], here we used another common K12-derived strain MG1655, where Δ*tyrA* and the aforementioned motility and chemotaxis mutations were introduced. Furthermore, since motility gene expression in *E. coli* is repressed by glucose [51], fructose was used instead as the carbon source. All knockout strains showed the expected motility phenotypes under these experimental growth conditions (Fig. S19). Moreover, no effects of fimbriation on swimming (Fig. S20) or of motility on the on/off state of the *fim* promoter (Fig. S21) were observed, confirming that motility and fimbriation do not exhibit cross-regulation under our experimental conditions.

Motility was observed to provide a significant competitive fitness benefit to *E. coli* at low initial cell densities and under conditions of mixing in an orbital shaker, with over 60% of the final *E. coli* partner population being wildtype for motility at the initial OD of 0.001 (Fig. 5B), which is comparable to typical microbial cell densities in aquatic environments [52]. This was true for competition with either *ΔmotA* or *ΔfliC* strains, suggesting that this effect is purely determined by motility, and not by possible flagella-mediated adhesion [53]. In contrast, chemotaxis does not seem to provide benefit under these conditions, since the fraction of a non-chemotactic but motile *ΔcheY* stain was not statistically different from 50% even at low initial cell density. The benefit of *E. coli* motility in the cross-feeding co-culture decreased and eventually inverted at lower shaking rates (Fig. 5B and Fig. S22), and both non-motile strains clearly outcompeted the motile one when the culture was grown in the absence of shaking, possibly as a consequence of co-sedimentation between non-motile *E. coli* and *S. cerevisiae* cells. The slight but significant increase in fitness of *ΔcheY* compared to the chemotactic strain in the absence of shaking could also be due to its known [54] increased residence time at the surface (Fig. S19B, G, H), and therefore more frequent encounters with sedimented yeast cells.

The beneficial effects of motility at high shaking rates required physical association between partners, since the beneficial effect of motility was no longer present when the competing strains were fimbrialess (Fig. 5C). It was also apparently related to cross-feeding, with the fraction of *ΔmotA* cells showing an average close to 50% once the community was grown in supplemented medium (Fig. 5D). Cross-feeding was also necessary to observe the beneficial effect of *ΔmotA* sedimentation or of *ΔcheY* surface trapping under static conditions. The motile partner was outcompeted by *ΔfliC* knockout in the absence of cross-feeding, which is consistent with the general growth advantage this strain has due to the absence of burden imposed by flagellar biosynthesis [51, 55]. However, this fitness cost of motility was not observed in the non-aggregating cross-feeding community, where the wildtype and *ΔfliC* strains maintained an equal ratio, indicating that under these conditions the growth is not limited by protein biosynthesis.

Motility of the *E. coli* partner also increased its competitiveness against a non-cooperator strain in a turbulent environment. When a motile cheater was introduced, in equal amounts with an *E. coli* partner, in co-cultures with yeast (Fig. 5E), motility of the partner modestly but significantly reduced cheater abundance when the culture was grown with shaking, but only when the partner was fimbriated. Consistent with the results above (Fig. 5B), the non-motile *ΔmotA* strain showed higher fitness in the absence of shaking, again likely due to the co-sedimentation with yeast. Comparable results were also obtained when the cheater was non-motile (Fig. S23A). The positive effect of partner motility was abolished when the media was supplemented with CSM, corroborating its dependence on cross-feeding (Fig. S23B).

## Discussion

Microbial communities relying on metabolite exchange have been previously studied using different mutants of the same species [56–58] or natural isolates [59, 60], either in a dispersed liquid culture [56] or under spatial assortment resulting from growth on a surface or in a microfluidic device [9, 15]. Here we investigated the impact of the direct physical association and co-aggregation between the cooperating partners and of the partner motility, which are frequently observed in natural suspended communities growing in turbulent aquatic environments [28, 61, 62], using an engineered mutualistic consortium between *S. cerevisiae* and *E. coli*.

We observed that physical association and co-aggregation, although moderately reducing the overall community growth, provides competitive fitness benefit to the bacterial partner. This effect is likely explained by the fact that proximity ensures preferential access of associated partners to secreted amino acids within co-aggregates. Proximity dependence of cross-feeding has been previously observed for communities grown on surfaces [26] or in microfluidic devices [9], where stable long-range metabolite gradients can be maintained. Here we demonstrate that the benefit of physical association can also be observed in a turbulent environment. Since our synthetic community is not likely to rely on a specific matrix that could retain secreted metabolites [26, 63–65] or on direct cytoplasmic channeling of metabolites, the efficiency of amino acid uptake by the yeast-associated *E. coli* appears to be sufficiently high to ensure that metabolites are locally consumed by the associated partners before dispersing into the environment. Physical association and formation of mixed aggregates might further enhance partner intermixing, which is generally important for mutualistic interactions [17].

Besides its immediate benefit to the bacterial partner, physical association partly protects the community as a whole against invasion by a non-cooperating bacterial cheater strain that consumes but does not share metabolites. Although under our conditions such a cheater establishes itself in the community at a relatively low frequency, it can nevertheless cause community collapse in semi-continuous co-culture. This is consistent with the theoretically predicted and experimentally observed tragedy of the commons [4–6, 66–68], and it contrasts with the coexistence between the partner and cheater described in previous studies [68–72]. We hypothesize that decrease in cheater abundance and delayed community collapse in the presence of the fimbriated bacterial partner result from faster growth of aggregates that contain fewer cheater cells, since the non-cooperating strain makes no contribution to the growth of the aggregate. Such effect, that has been previously theorized [73] and described as analogous to the Simpsons paradox [74], leads to the overall decrease in the cheater abundance in the community. This beneficial protective effect of co-aggregation on the community in the presence of a non-cooperator overweighs its cost for the community growth.

These fitness advantages of fimbriation in suspended co-culture are no longer observed when communities are grown on a solid medium, likely because the segregation between *E. coli* strains provided by the spatial structure of the colony [16] reduces their direct competition and ensures that resources are only shared locally even without direct physical association between partners. This segregation may also reduce local growth of non-cooperating community members, as already reported previously [10], and we indeed observed a lower fraction of cheater cells within the colony community than in the non-aggregating liquid culture.

In a turbulent environment, interacting partners could further profit from motility, which allows them to outcompete the non-motile partners and to reduce invasion by a cheater strain. Consistent with previous studies [51], although flagella biosynthesis showed a pronounced burden on *E. coli* growth in supplemented medium, we observed that motility has a net benefit under cross-feeding conditions. This advantage of motility is likely explained by the increased encounter rate and therefore of association between fimbriated bacteria and yeast cells that are kept in suspension by mixing, as previously described for interactions between motile bacteria and suspended particles [75, 76]. In aquatic environments swimming remains faster than stirring at the spatial scales below 0.1–1 mm [77], meaning that motility can enhance local cell encounters even in a well-mixed culture. Consistent with that, motility provided an advantage only at low initial cell densities, comparable to the ones observed in aquatic environments [52], where locating partners is particularly challenging. An additional enhancement of attachment might result from the non-homogeneous distribution of motile organisms in the presence of turbulence [78, 79]. In contrast, in the static co-culture non-motile bacteria might have an advantage regardless of physical association, because of their co-sedimentation with yeast and thus closer proximity to the partner in the sessile community of the sediment.

In summary, we demonstrate that direct physical association and partner motility can provide fitness benefits to one or both partners in the mutualistic community growing in a turbulent environment, which outweigh their costs at low initial density of the co-culture and in the presence of non-cooperators. Since low densities and the presence of competitors are likely to be common in aquatic environments, we propose that these benefits might explain the widespread presence of mechanisms involved in cell-cell adhesion and motility in natural pelagic communities [27, 28].

## Supplementary information

Supplementary Information

## Data Availability

All data reported in this paper will be shared by the lead contact upon request.

## References

[1] D’Souza G, Shitut S, Preussger D, Yousif G, Waschina S, Kost C (2018). Ecology and evolution of metabolic cross-feeding interactions in bacteria. Nat Prod Rep.

[2] López-García P, Moreira D (2020). The Syntrophy hypothesis for the origin of eukaryotes revisited. Nat Microbiol.

[3] Phelan VV, Liu W-T, Pogliano K, Dorrestein PC (2012). Microbial metabolic exchange—the chemotype-to-phenotype link. Nat Chem Biol.

[4] Smith P, Schuster M (2019). Public goods and cheating in microbes. Curr Biol.

[5] Özkaya Ö, Balbontín R, Gordo I, Xavier KB (2018). Cheating on cheaters stabilizes cooperation in *Pseudomonas aeruginosa*. Curr Biol.

[6] Schuster M, Foxall E, Finch D, Smith H, De Leenheer P (2017). Tragedy of the commons in the chemostat. PLoS ONE.

[7] Welch JLM, Rossetti BJ, Rieken CW, Dewhirst FE, Borisy GG (2016). Biogeography of a human oral microbiome at the micron scale. Proc Natl Acad Sci USA.

[8] Pernthaler A, Dekas AE, Brown CT, Goffredi SK, Embaye T, Orphan VJ (2008). Diverse syntrophic partnerships from deep-sea methane vents revealed by direct cell capture and metagenomics. Proc Natl Acad Sci USA.

[9] Dal CoA, van Vliet S, Kiviet DJ, Schlegel S, Ackermann M (2020). Short-range interactions govern the dynamics and functions of microbial communities. Nat Ecol Evol.

[10] Pande S, Kaftan F, Lang S, Svato A, Germerodt S, Kost C (2016). Privatization of cooperative benefits stabilizes mutualistic cross-feeding interactions in spatially structured environments. ISME J.

[11] Momeni B, Waite AJ, Shou W (2013). Spatial self-organization favors heterotypic cooperation over cheating. Elife.

[12] Campbell K, Vowinckel J, Mülleder M, Malmsheimer S, Lawrence N, Calvani E (2015). Self-establishing communities enable cooperative metabolite exchange in a eukaryote. Elife.

[13] Chen F, Wegner SV (2020). Blue-light-switchable bacterial cell-cell adhesions enable the control of multicellular bacterial communities. ACS Synth Biol.

[14] Müller MJI, Neugeboren BI, Nelson DR, Murray AW (2014). Genetic drift opposes mutualism during spatial population expansion. Proc Natl Acad Sci USA.

[15] Momeni B, Brileya KA, Fields MW, Shou W (2013). Strong inter-population cooperation leads to partner intermixing in microbial communities. Elife.

[16] Blanchard AE, Lu T (2015). Bacterial social interactions drive the emergence of differential spatial colony structures. BMC Syst Biol.

[17] Kovács ÁT (2014). Impact of spatial distribution on the development of mutualism in microbes. Front Microbiol.

[18] Harcombe WR, Chacón JM, Adamowicz EM, Chubiz LM, Marx CJ (2018). Evolution of bidirectional costly mutualism from byproduct consumption. Proc Natl Acad Sci USA.

[19] Preussger D, Giri S, Muhsal LK, Oña L, Kost C (2020). Reciprocal fitness feedbacks promote the evolution of mutualistic cooperation. Curr Biol.

[20] Marchal M, Goldschmidt F, Derksen-Müller SN, Panke S, Ackermann M, Johnson DR (2017). A passive mutualistic interaction promotes the evolution of spatial structure within microbial populations. BMC Evol Biol.

[21] Mehta AP, Supekova L, Chen J-H, Pestonjamasp K, Webster P, Ko Y (2018). Engineering yeast endosymbionts as a step toward the evolution of mitochondria. Proc Natl Acad Sci USA.

[22] Karkar S, Facchinelli F, Price DC, Weber APM, Bhattacharya D (2015). Metabolic connectivity as a driver of host and endosymbiont integration. Proc Natl Acad Sci USA.

[23] Mergaert P, Kikuchi Y, Shigenobu S, Nowack ECM (2017). Metabolic integration of bacterial endosymbionts through antimicrobial peptides. Trends Microbiol.

[24] Pande S, Shitut S, Freund L, Westermann M, Bertels F, Colesie C (2015). Metabolic cross-feeding via intercellular nanotubes among bacteria. Nat Commun.

[25] Shitut S, Ahsendorf T, Pande S, Egbert M, Kost C (2019). Nanotube-mediated cross-feeding couples the metabolism of interacting bacterial cells. Environ Microbiol.

[26] Nadell CD, Drescher K, Foster KR (2016). Spatial structure, cooperation and competition in biofilms. Nat Rev Microbiol.

[27] Rickard AH, Gilbert P, High NJ, Kolenbrander PE, Handley PS (2003). Bacterial coaggregation: an integral process in the development of multi-species biofilms. Trends Microbiol.

[28] Steffan BN, Venkatesh N, Keller NP (2020). Let’s get physical: bacterial-fungal interactions and their consequences in agriculture and health. J Fungi.

[29] Schweitzer-Natan O, Ofek-Lalzar M, Sher D, Sukenik A (2019). Particle-associated microbial community in a subtropical lake during thermal mixing and phytoplankton succession. Front Microbiol.

[30] Cai YM (2020). Non-surface attached bacterial aggregates: a ubiquitous third lifestyle. Front Microbiol.

[31] Monteil CL, Vallenet D, Menguy N, Benzerara K, Barbe V, Fouteau S (2019). Ectosymbiotic bacteria at the origin of magnetoreception in a marine protist. Nat Microbiol.

[32] Husnik F, Tashyreva D, Boscaro V, George EE, Lukeš J, Keeling PJ (2021). Bacterial and archaeal symbioses with protists. Curr Biol.

[33] Müller J, Overmann J (2011). Close Interspecies Interactions between Prokaryotes from sulfureous environments. Front Microbiol.

[34] Overmann J, van Gemerden H (2000). Microbial interactions involving sulfur bacteria: implications for the ecology and evolution of bacterial communities. FEMS Microbiol Rev.

[35] Johnson WM, Alexander H, Bier RL, Miller DR, Muscarella ME, Pitz KJ (2020). Auxotrophic interactions: a stabilizing attribute of aquatic microbial communities?. FEMS Microbiol Ecol.

[36] Colin R, Ni B, Laganenka L, Sourjik V (2021). Multiple functions of flagellar motility and chemotaxis in bacterial physiology. FEMS Microbiol Rev.

[37] Raina J-B, Fernandez V, Lambert B, Stocker R, Seymour JR (2019). The role of microbial motility and chemotaxis in symbiosis. Nat Rev Microbiol.

[38] Robinson CD, Sweeney EG, Ngo J, Ma E, Perkins A, Smith TJ (2021). Host-emitted amino acid cues regulate bacterial chemokinesis to enhance colonization. Cell Host Microbe.

[39] Konopka A (2009). What is microbial community ecology?. ISME J.

[40] Jann K, Schmidt G, Blumenstock E, Vosbeck K (1981). *Escherichia coli* adhesion to *Saccharomyces cerevisiae* and mammalian cells: role of piliation and surface hydrophobicity. Infect Immun.

[41] Blattner FR, Plunkett G, Bloch CA, Perna NT, Burland V, Riley M (1997). The complete genome sequence of *Escherichia coli* K-12. Science.

[42] Jensen SI, Lennen RM, Herrgård MJ, Nielsen AT (2016). Seven gene deletions in seven days: Fast generation of *Escherichia coli* strains tolerant to acetate and osmotic stress. Sci Rep.

[43] Giaever G, Chu AM, Ni L, Connelly C, Riles L, Véronneau S (2002). Functional profiling of the *Saccharomyces cerevisiae* genome. Nature.

[44] Abraham JM, Freitag CS, Clements JR, Eisenstein BI (1985). An invertible element of DNA controls phase variation of type 1 fimbriae of *Escherichia coli*. Proc Natl Acad Sci USA.

[45] Dubey GP, Ben-Yehuda S (2011). Intercellular nanotubes mediate bacterial communication. Cell.

[46] Hallatschek O, Hersen P, Ramanathan S, Nelson DR (2007). Genetic drift at expanding frontiers promotes gene segregation. Proc Natl Acad Sci USA.

[47] D’Souza G, Waschina S, Pande S, Bohl K, Kaleta C, Kost C (2014). Less is more: selective advantages can explain the prevalent loss of biosynthetic genes in bacteria. Evolution.

[48] Wang M, Schaefer AL, Dandekar AA, Greenberg EP (2015). Quorum sensing and policing of *Pseudomonas aeruginosa* social cheaters. Proc Natl Acad Sci USA.

[49] Greig D, Travisano M (2004). The Prisoner’s Dilemma and polymorphism in yeast SUC genes. Proc R Soc Lond Ser B Biol Sci.

[50] Parker DJ, Demetci P, Li GW (2019). Rapid accumulation of motility-activating mutations in resting liquid culture of *Escherichia coli*. J Bacteriol.

[51] Ni B, Colin R, Link H, Endres RG, Sourjik V (2020). Growth-rate dependent resource investment in bacterial motile behavior quantitatively follows potential benefit of chemotaxis. Proc Natl Acad Sci USA.

[52] Whitman WB, Coleman DC, Wiebe WJ (1998). Prokaryotes: the unseen majority. Proc Natl Acad Sci USA.

[53] Friedlander RS, Vogel N, Aizenberg J (2015). Role of flagella in adhesion of *Escherichia coli* to abiotic surfaces. Langmuir.

[54] Suchanek VM, Esteban‐López M, Colin R, Besharova O, Fritz K, Sourjik V (2020). Chemotaxis and cyclic‐di‐GMP signalling control surface attachment of *Escherichia coli*. Mol Microbiol.

[55] Ni B, Ghosh B, Paldy FS, Colin R, Heimerl T, Sourjik V (2017). Evolutionary remodeling of bacterial motility checkpoint control. Cell Rep.

[56] Wintermute EH, Silver PA (2010). Emergent cooperation in microbial metabolism. Mol Syst Biol.

[57] Shou W, Ram S, Vilar JMG (2007). Synthetic cooperation in engineered yeast populations. Proc Natl Acad Sci USA.

[58] Pande S, Merker H, Bohl K, Reichelt M, Schuster S, Figueiredo LFde (2014). Fitness and stability of obligate cross-feeding interactions that emerge upon gene loss in bacteria. ISME J.

[59] Koo H, Andes DR, Krysan DJ (2018). *Candida*–streptococcal interactions in biofilm-associated oral diseases. PLoS Pathog.

[60] Stadie J, Gulitz A, Ehrmann MA, Vogel RF (2013). Metabolic activity and symbiotic interactions of lactic acid bacteria and yeasts isolated from water kefir. Food Microbiol.

[61] Cordero OX, Datta MS (2016). Microbial interactions and community assembly at microscales. Curr Opin Microbiol.

[62] Grossart HP, Riemann L, Azam F (2001). Bacterial motility in the sea and its ecological implications. Aquat Micro Ecol.

[63] Emge P, Moeller J, Jang H, Rusconi R, Yawata Y, Stocker R (2016). Resilience of bacterial quorum sensing against fluid flow. Sci Rep.

[64] Flemming H-C, Wingender J, Szewzyk U, Steinberg P, Rice SA, Kjelleberg S (2016). Biofilms: an emergent form of bacterial life. Nat Rev Microbiol.

[65] Drescher K, Nadell CD, Stone HA, Wingreen NS, Bassler BL (2014). Solutions to the public goods dilemma in bacterial biofilms. Curr Biol.

[66] Yan H, Wang M, Sun F, Dandekar AA, Shen D, Li N (2018). A metabolic trade-off modulates policing of social cheaters in populations of *Pseudomonas aeruginosa*. Front Microbiol.

[67] Cavaliere M, Yang G, Danos V, Dakos V (2016). Detecting the collapse of cooperation in evolving networks. Sci Rep.

[68] Gore J, Youk H, Van, Oudenaarden A (2009). Snowdrift game dynamics and facultative cheating in yeast. Nature.

[69] Bastiaans E, Debets AJM, Aanen DK (2016). Experimental evolution reveals that high relatedness protects multicellular cooperation from cheaters. Nat Commun.

[70] Velicer GJ, Kroos L, Lenski RE (2000). Developmental cheating in the social bacterium *Myxococcus xanthus*. Nature.

[71] Moreno-Fenoll C, Cavaliere M, Martínez-García E, Poyatos JF (2017). Eco-evolutionary feedbacks can rescue cooperation in microbial populations. Sci Rep.

[72] Sanchez A, Gore J (2013). Feedback between population and evolutionary dynamics determines the fate of social microbial populations. PLoS Biol.

[73] Wilson DS (1975). A theory of group selection. Proc Natl Acad Sci USA.

[74] Chuang JS, Rivoire O, Leibler S (2009). Simpson’s Paradox in a synthetic microbial system. Science.

[75] Lambert BS, Fernandez VI, Stocker R (2019). Motility drives bacterial encounter with particles responsible for carbon export throughout the ocean. Limnol Oceanogr Lett.

[76] Schauer O, Mostaghaci B, Colin R, Hürtgen D, Kraus D, Sitti M (2018). Motility and chemotaxis of bacteria-driven microswimmers fabricated using antigen 43-mediated biotin display. Sci Rep.

[77] Taylor JR, Stocker R (2012). Trade-offs of chemotactic foraging in turbulent water. Science.

[78] Rusconi R, Guasto JS, Stocker R (2014). Bacterial transport suppressed by fluid shear. Nat Phys.

[79] Durham WM, Climent E, Barry M, De Lillo F, Boffetta G, Cencini M (2013). Turbulence drives microscale patches of motile phytoplankton. Nat Commun.

