# Supplementary material for: Impact of direct physical association and motility on fitness of a synthetic interkingdom microbial community
Source: ISME J. 2022 Dec 24;17(3):371–81. doi: 10.1038/s41396-022-01352-2 (PMC9938286; doi:10.1038/s41396-022-01352-2)
Supplement: Supplementary file 1 — Supplementary Information [file 41396_2022_1352_MOESM1_ESM.pdf]

Supplementary Information

Impact of direct physical association and motility on fitness of a synthetic interkingdom microbial community

Giovanni Scarinci and Victor Sourjik

[1]Max Planck Institute for Terrestrial Microbiology and Center for Synthetic Microbiology (SYNMIKRO), Marburg, Germany

*Corresponding author:

Prof. Dr. Victor Sourjik
Max Planck Institute for Terrestrial Microbiology and Center for Synthetic Microbiology (SYNMIKRO)
Department of Systems and Synthetic Microbiology
Karl-von-Frisch-Str. 14, D-35043 Marburg, Germany
victor.sourjik@synmikro.mpi-marburg.mpg.de

Supplementary Materials and Methods

Aggregation assay

E. coli and *S. cerevisiae* cultures were grown as described in materials and methods. Cells were washed twice with PBS and resuspended in 1 mL PBS. After that, bacterial and yeast cells were mixed at a final OD_{600} 0.5 for *S. cerevisiae* and 0.2 for *E. coli* and incubated for 1 h at room temperature in a 24-well plate with shaking (200 r.p.m.) prior to microscopy imaging.

fim promoter orientation assay

In order to verify the orientation of the fimbriae operon (*fim*), a procedure similar to the one described in [1] was followed. Specifically, co-cultures were grown as described in the growth conditions section for 72 h, followed by genome extraction using the DNeasy Blood & Tissue Kit (Quiagen, Hilden, Germany). From this, 1 µL was used as PCR template using the Q5 polymerase (New England Biolabs, Frankfurt am Main, Germany) and with primers P1 (5'-AGTAATGCTGCTCGTTTTGC-3') and P2 (5'-GCTGTAGAACTGAGGGACAG-3'). PCR products were then purified (Zymo research Europe GMBH, Freiburg, Germany) and digested for 2 h with *Sna*BI (New England Biolabs, Frankfurt am Main, Germany). Subsequently, samples were separated using gel electrophoresis in a 2% agarose. Band intensity analysis were performed using ImageJ [2].

Cell tracking

Movies were acquired with a phase contrast microscope at 10x magnification (NA = 0.3) and a Mikrotron Eosens camera (1 px = 0.7 µm) running at 50 frames per seconds (fps) for 2000 frames. Image analysis, Z-stack projections, particle tracking and image correlation analysis were performed using ImageJ [2] and custom-made algorithms run as plugins in ImageJ. As previously described [3], the radius of gyration $R_i = \langle \left(\left(r_i(t) \right) - \left(r_i(t) \right)_t \right)^2 \rangle_t / T$ was used to sort swimmers from non-swimmers and determine the fraction of motile cells. Tracking data from swimmer cells were then analyzed to calculate tumbling rate, swimming speed and average residence time.

Statistical analysis

Both the statistical tests used and sample size (n) are specified in the figure legends. In all cases, n refers to the number of independent co-cultures derived from independent precultures of single strains. Technical replicates are defined as independent co-cultures derived from the same precultures of single strains. For pairwise comparisons asterisks indicates statistical difference (*p<0.05, **p<0.01, ***p<0.001, ****p<0.0001). For boxplots, the internal line indicates the median of values, while the regions of the box below and above indicate respectively the 25th and 75th percentiles. Whiskers extend up to 1.5x the interquartile range from the 25th to the 75th percentile. For line plots, data is displayed as mean values ± S.D. confidence interval. For scatter plots, central black bars represent the

mean value and the whiskers extend to ± S.D as confidential interval, while circles indicate the biological replicates. Statistical analyses (*t*-test one- and two-way ANOVA) were performed using either JupyterLab (ANACONDA) or Microsoft Excel. Correlation analysis were performed using the data analysis add-in of Microsoft Excel and plotted using the regplot function of the seaborn package JupyterLab (ANACONDA). The shadow part represents 95% confidence interval while the lines represent the linear regression fit. Mean growth rate values for the specific time interval were calculated as the difference between the log_2 of the OD600 measured respectively at 25 h and 16 h divided by the time interval expressed in hours.

Supplementary Figures

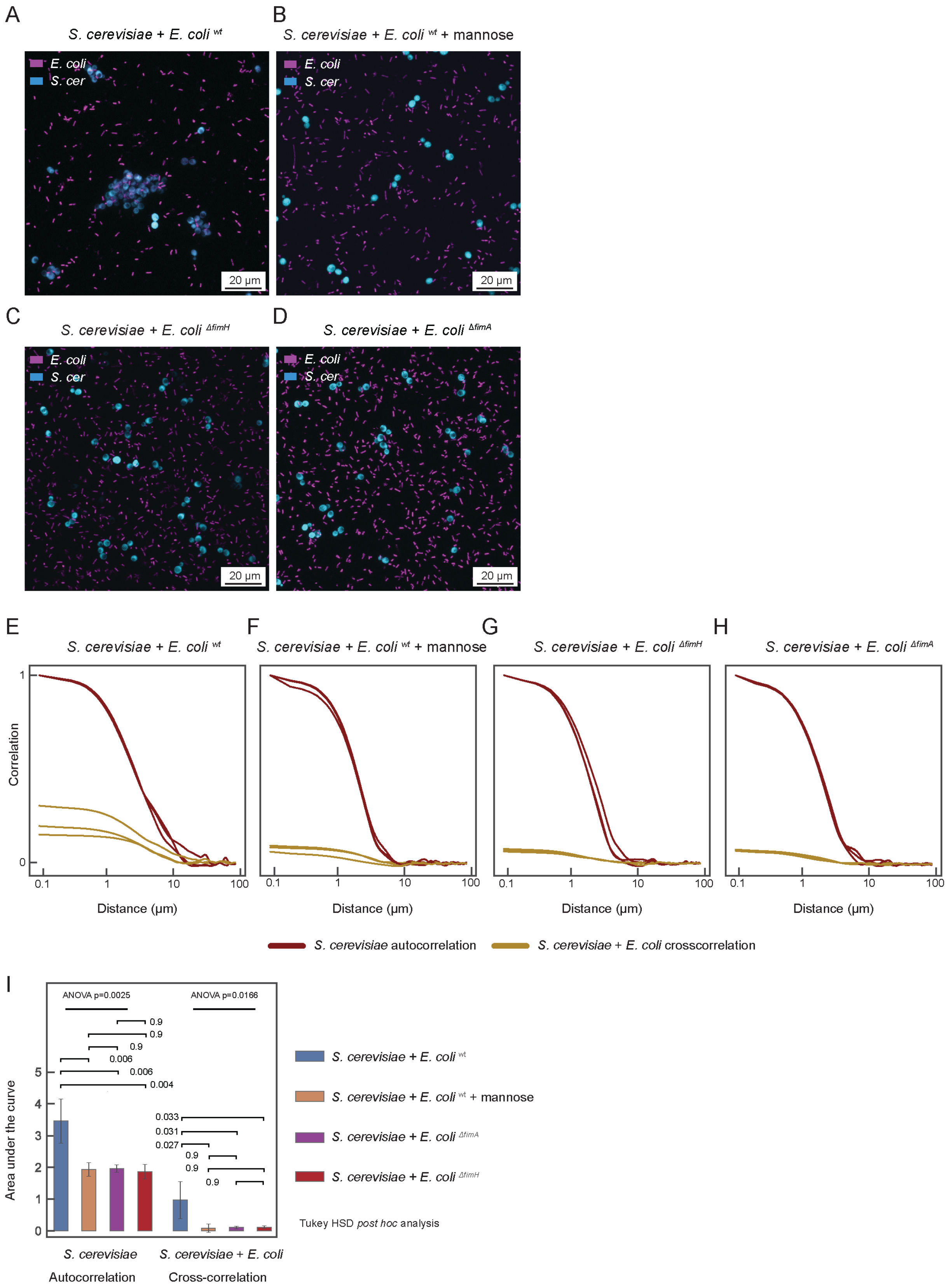

Figure S1. Characterization of *S. cerevisiae* and *E. coli* co-aggregation

A-D Confocal microscopy images of mixtures of *S. cerevisiae* expressing mTurquoise2 (blue) with *E. coli* that is (A) Fim$^+$ (wt), (B) Fim$^+$ in presence of 4% mannose, or lacking either (C) the tip (Δ*fimH)* of the mannose-binding fimbriae filaments or (D) the entire filament (Δ*fimA*). In all cases *E. coli* (magenta) is expressing mCherry. Scale bar = 20 µm. **E-H** Auto- and cross-correlation analysis of co-aggregation between *S. cerevisiae* and (E) *E. coli* Fim$^+$, (F) *E. coli* Fim$^+$ in presence of mannose, (G) *E. coli* Δ*fimH* and (H) *E. coli* Δ*fimA*, each with three biological replicates (represented by different lines). Autocorrelation analysis between neighboring pixels in one fluorescent channel (mTurquoise2) reflects the characteristic size of yeast cells or/and aggregates, whereas the cross-correlation analysis between two different channels (mCherry and mTurquoise2) reflects the characteristic size and number of mixed bacteria-yeast aggregates. These analyses were performed for the entire images, with each image contained at least twenty yeast cells and one hundred bacterial cells. **I** Quantification of aggregation, calculated as area under the curve for auto- and cross-correlations analysis from the plots shown in E-H. One-way ANOVA tests, followed by an HSD Tukey test as *post hoc* analysis were performed from three biological replicates.

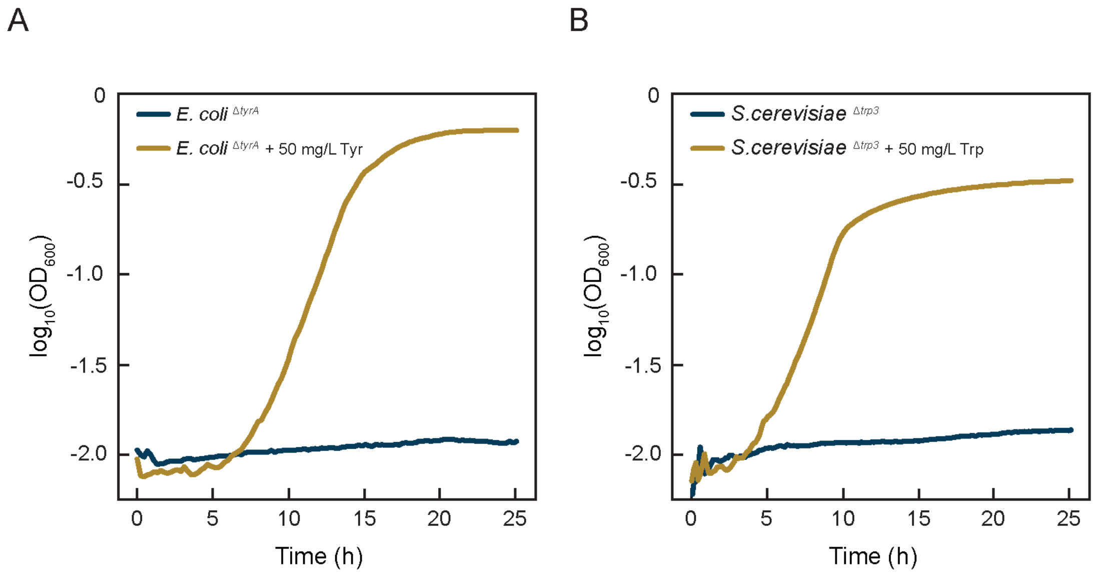

Figure S2. Verification of auxotrophies

A,B Growth curves of monocultures of (A) *E. coli* Δ*tyrA* and (B) *S. cerevisiae* Δ*trp3* in minimal media either not supplemented (blue lines) or supplemented with the required amino acid (yellow lines).

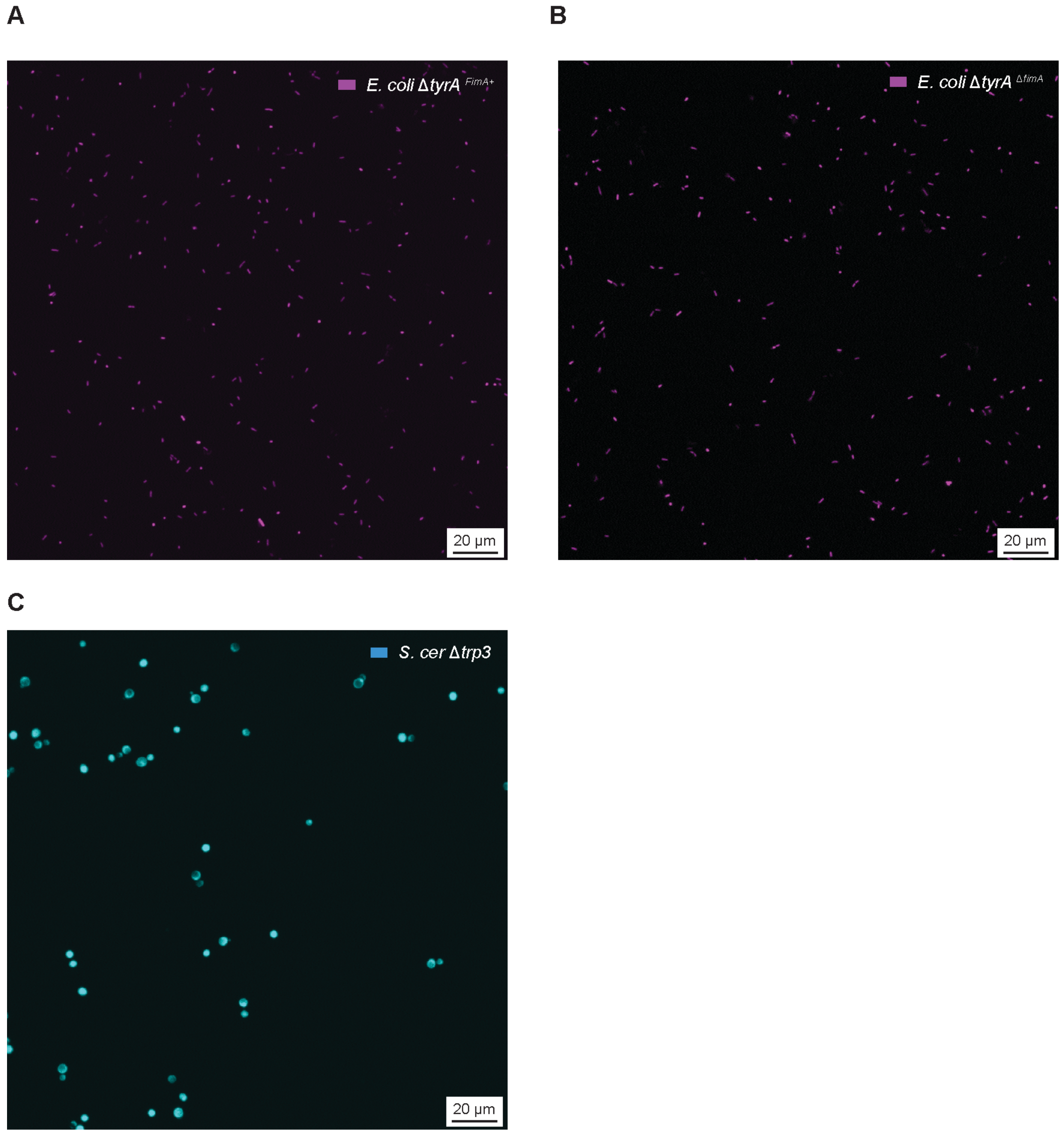

Figure S3. Absence of aggregation in monocultures

A-C Confocal microscopy images of single cultures of (A) *E. coli* FimA⁺ Δ*tyrA*, (B) *E. coli* Δ*fimA* Δ*tyrA,* both expressing mCherry (magenta), or (C) *S. cerevisiae* Δ*trp3* expressing mTurquoise2 (blue) grown in YNB glucose supplemented with CSM. Scale bar = 20 µm.

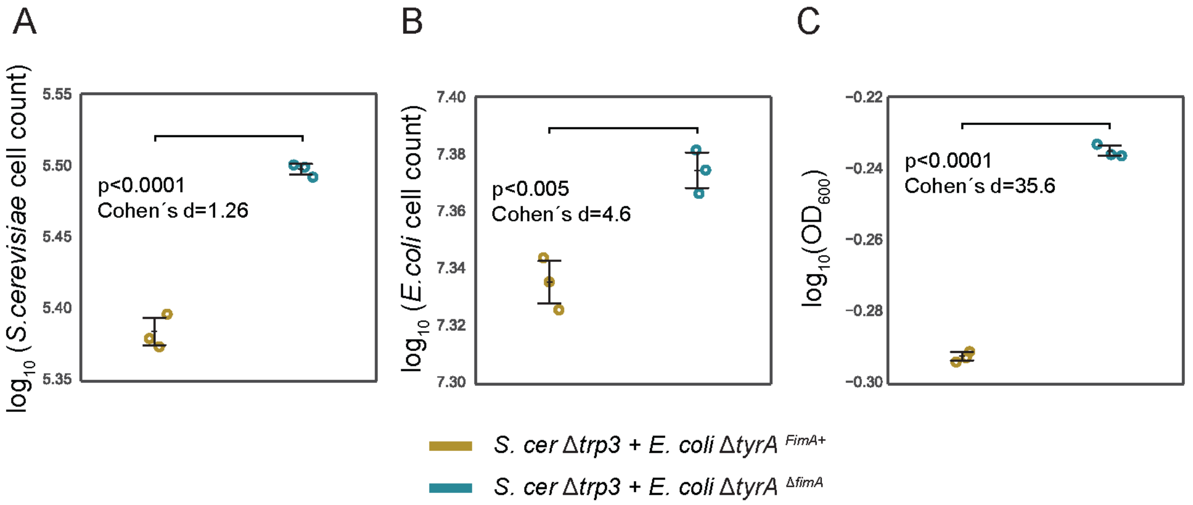

Figure S4. Growth of partners in cross-feeding communities with or without adhesion

A,B Final cell counts from co-cultures as in Figure 2A,B for (A) *S. cerevisiae* and (B) *E. coli*. *p* values were obtained from a two-tailed *t*-tests assuming equal variances of the data sets, each with three biological replicates (indicated by circles). Cohen´s *d* values were calculated to quantify the effect size. **C** Final OD from co-cultures in Figure 2A,B. *p* values were obtained from a two-tailed *t*-tests assuming equal variances of the data sets, each with three biological replicates (indicated as circles). Cohen´s *d* values were calculated to quantify the effect size.

A

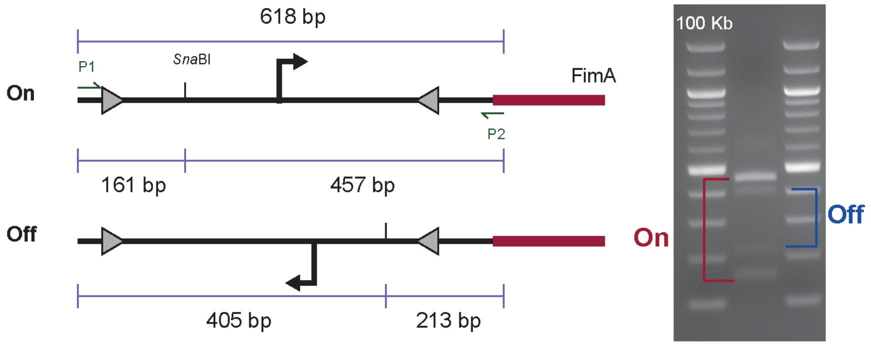

B

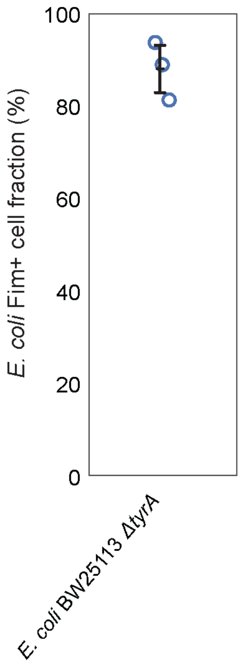

Figure S5. Activation status of the *fim* operon

A Assay used to determine the promoter orientation. The promoter region (618 bp) of the *fim* operon was amplified by PCR using primers P1 and P2, as indicated. This region contains a unique *Sna*BI restriction site. The digestion of the PCR fragments with *Sna*BI results in specific fragment pairs according to the state (On/Off) of the promoter, thus displaying a specific pattern of bands once the digestion is run via electrophoresis on a 2% agarose gel. **B** Quantification of the *fim* status of the *E. coli* partner based on band intensities as shown in (A), which is comparable to values obtained in LB cultures. Three biological replicates were used and are indicated as circles.

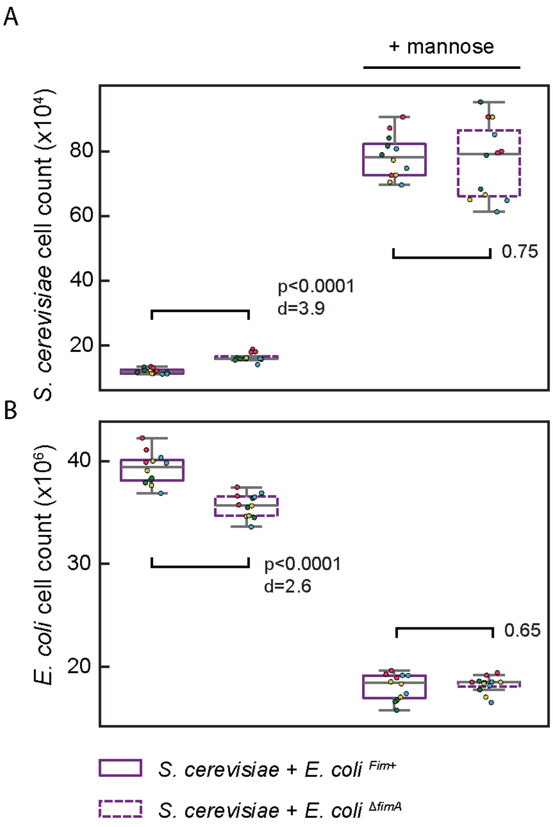

Figure S6. Effects of partner adhesion on growth

A,B Numbers of (A) *S. cerevisiae* and (B) *E. coli* cells measured by flow cytometry in co-cultures grown for 72 h in YNB-glucose supplemented with CSM either in absence or presence of 4% mannose, as indicated. Boxes represent the second and third quartile of the distribution and whiskers extends to show the rest of the distribution of four biological replicates (indicated by dots, different colors represent technical replicates for the same biological replica). *p* values from two-tailed *t*-tests assuming equal variances of the data sets. Cohen´s *d* values were calculated to quantify the effect size.

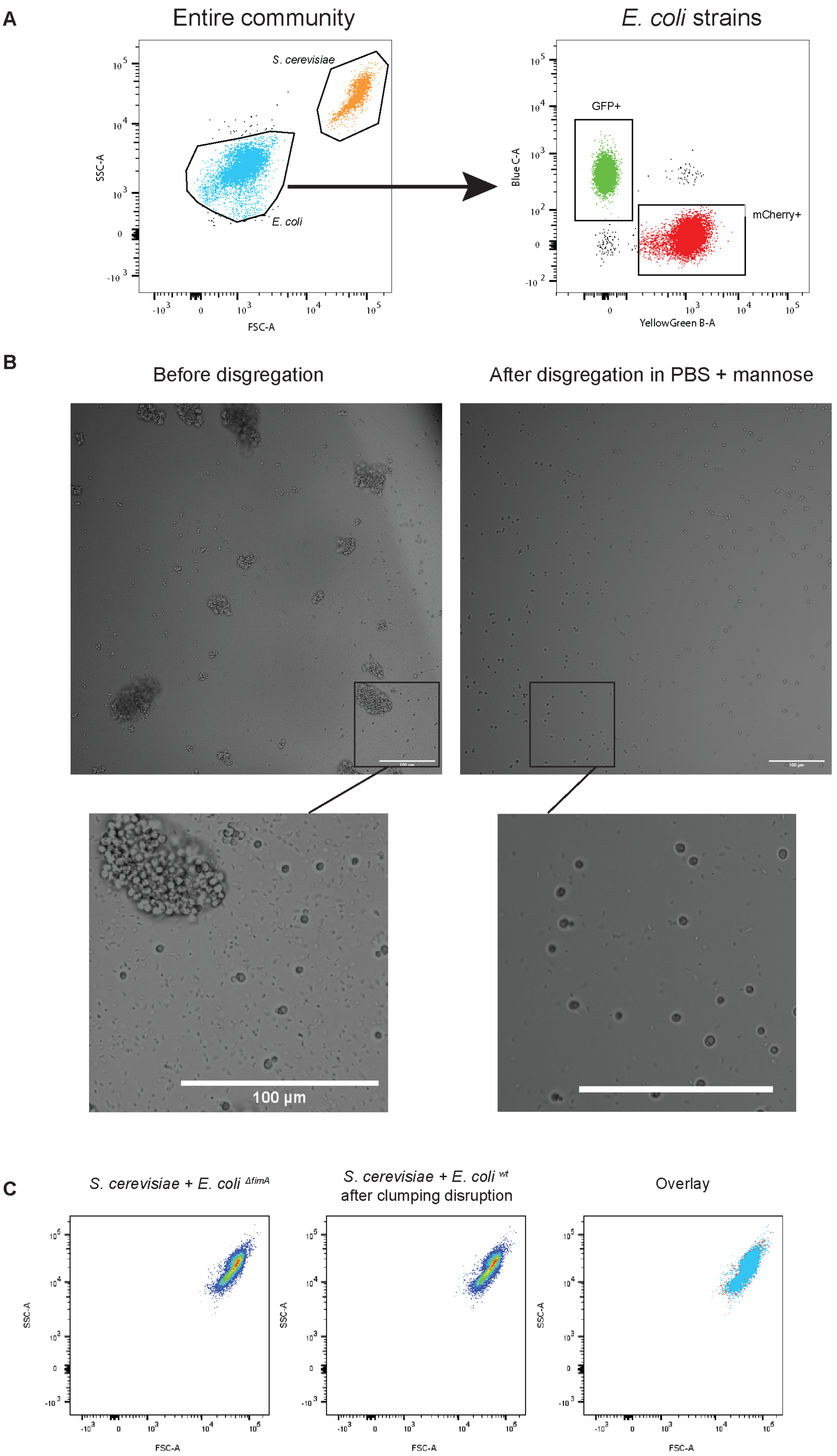

Figure S7. Flow cytometry measurements after cell aggregate disruption.

A Illustration of the gating strategy performed on flow cytometry data for *S. cerevisiae*-*E. coli* co-cultures. *E. coli* and *S. cerevisiae* were distinguished according to their different scatter properties (SSC and FSC). When

applicable, individual *E. coli* strains were further distinguished according to their respective fluorescent markers (Blue positive cells express GFP, YellowGreen positive cells express mCherry). **B** Microscopy images showing the complete disruption of aggregates after vigorous mixing in PBS + mannose. **C** Flow cytometry analysis performed on the gated *S. cerevisiae* subpopulation showing the overlap between a yeast population obtained after clump disruptions from an aggregative community and a population of *S. cerevisiae* grown with a fimbrialess bacterial partner, confirming complete disruption of the aggregates.

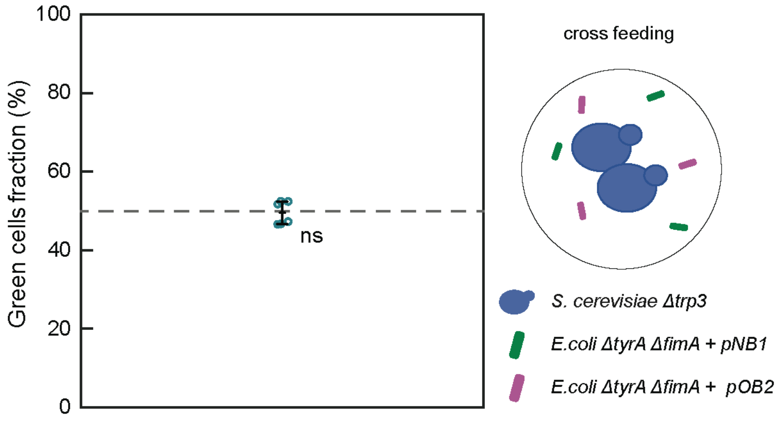

Figure S8. Relative growth fitness effects of different fluorescent markers

Final cell fraction of (sfGFP+) of *E. coli* cells in co-cultures with *S.cerevisiae* Δ*trp3* that were inoculated with equal amounts of *E. coli* Δ*tyrA* Δ*fimA* either expressing mCherry (magenta-pOB2) or sfGFP (green-pNB1) and grown in YNB glucose minimal media for 72 h. One sample *t*-test assessing for the difference from a 50% mean performed with six biological replicates represented as circles.

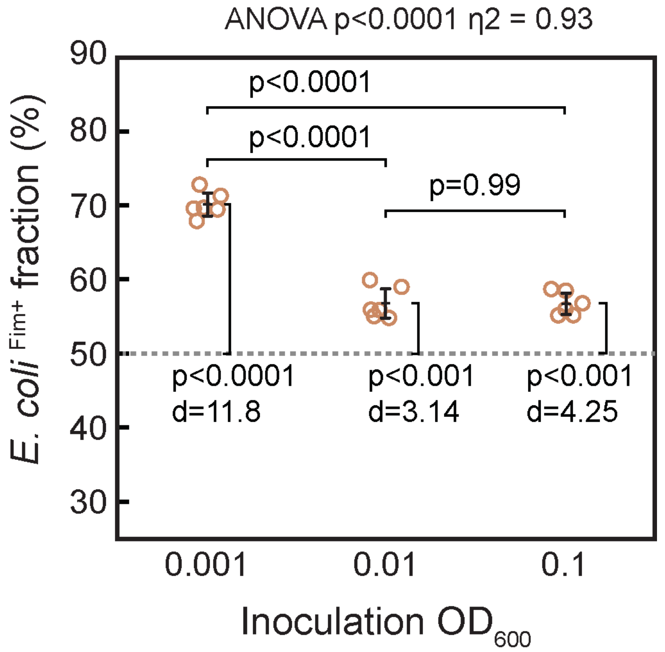

Figure S9. Effect of initial cell density on fitness benefit of *E. coli* fimbriation

Fraction of Fim+ cells (labeled with mCherry) in the total *E. coli* population measured by flow cytometry in co-culture with sfGFP-labelled fimbrialess (Δ*fimA*) *E. coli* and *S. cerevisiae* Δ*trp3.* Co-cultures were inoculated at different initial cell densities, with initially equal amounts of Fim^+ (labelled with mCherry) and Δ*fimA* (labelled with sfGFP) cells and grown for 96 h in YNB-glucose. Error bars represent standard deviations of six biological replicates represented as circles. One-way ANOVA test, followed by an HSD Tukey test as *post hoc* analysis were performed to assess for difference between samples. A one-sample *t*-test was performed to assess differences from an average fraction of 50%. Cohen´s *d* values were used to quantify the effect size.

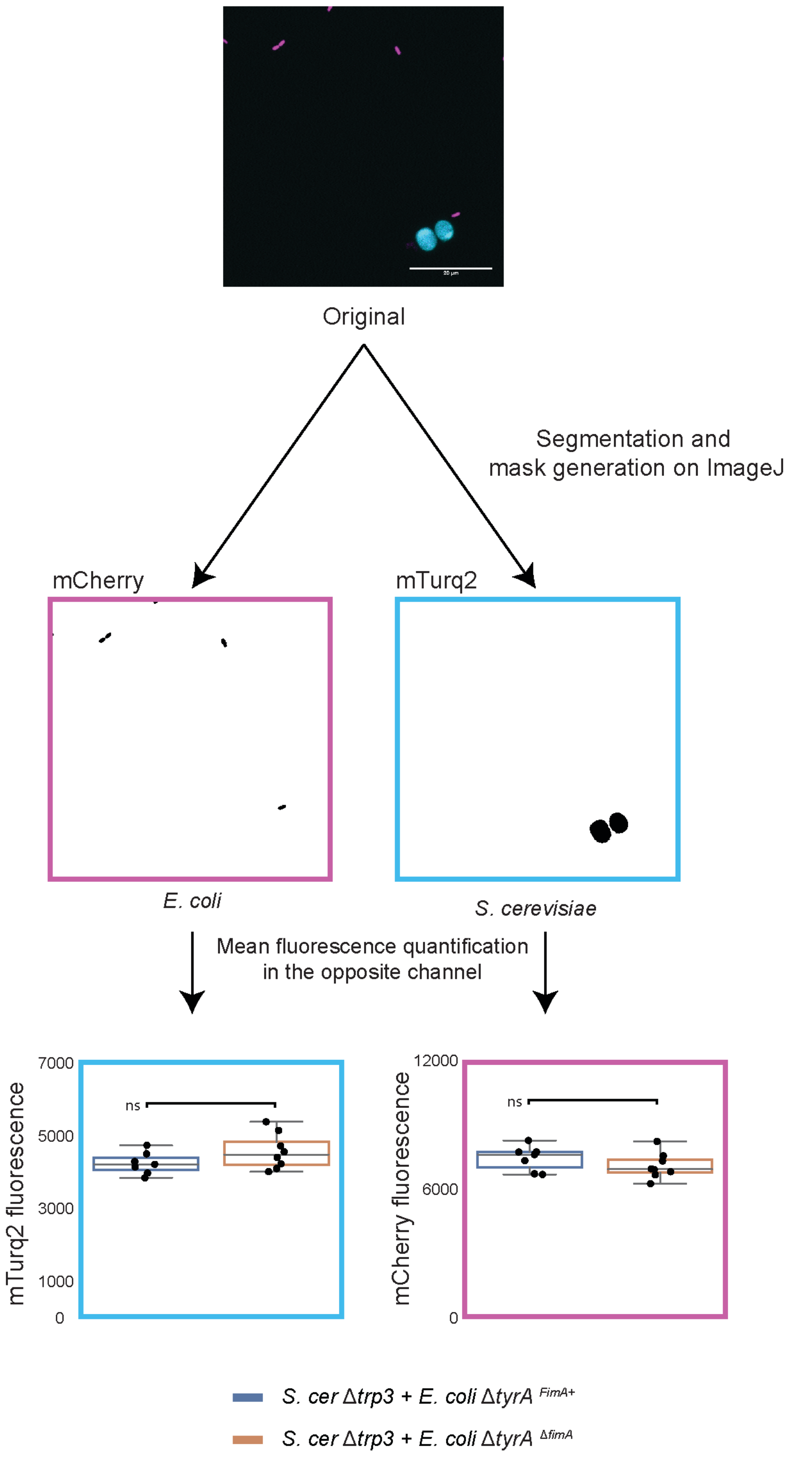

Figure S10. Test for direct cytoplasmic material exchange between partners

Following cell imaging and the ensuing segmentation to distinguish the two partners, the intensity in the channel corresponding to the fluorescence of the other partner was measured in the area of each organism. Blue and orange box plots in the lower panel represent samples from clumping or non-clumping communities respectively. In total, thirty-five yeast cells have been measured per each condition and above one-hundred for *E. coli*. *ns* from a two-tailed *t*-test assuming equal variances of the data sets represented by more than six biological replicates.

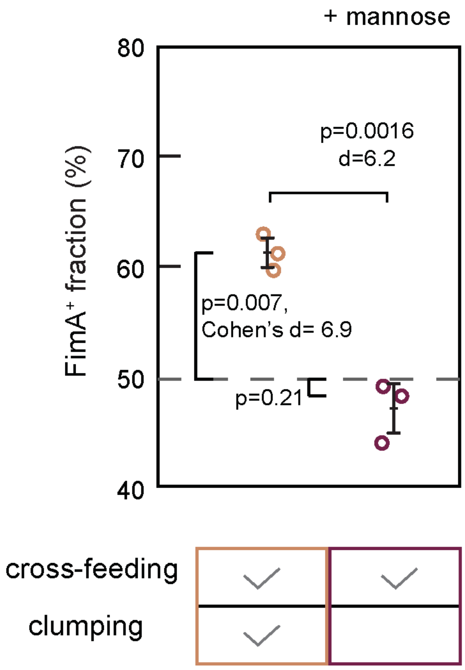

Figure S11. Effects of partner adhesion on fitness for a community with *S. cerevisiae* Δ*trp4* strain

Fraction of Fim$^+$ cells (labelled with mCherry) in the total *E. coli* population co-cultured with Δ*fimA E. coli* (labelled with sfGFP) and *S. cerevisiae* Δ*trp4* (labelled with mTurquoise2). Co-cultures were inoculated with equal amounts of Fim$^+$ and Δ*fimA* cells and grown for 96 h either in YNB-glucose or in YNB-glucose supplemented with 4% mannose. Scatter plots represent the distribution of three biological replicates (indicated by circles). Whiskers represent the standard deviation. Both two-tailed *t*-test assuming equal variances of the data sets and one sample *t*-test to assess differences from a 50% average were performed. Cohen´s *d* values were used to quantify the effect size.

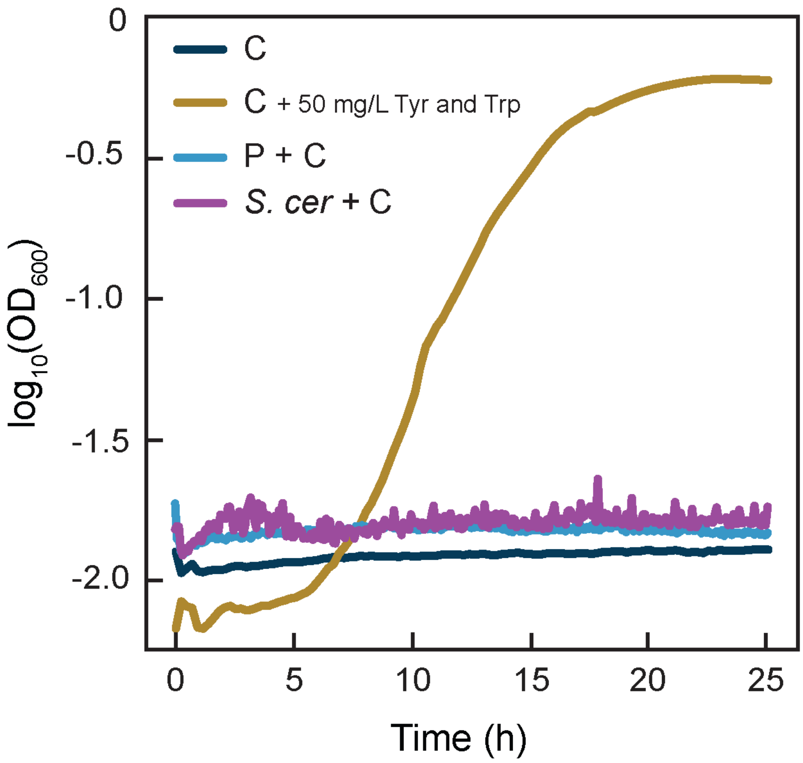

Figure S12. Verification of the auxotrophies

Growth curves from cultures of the *E. coli* Δ*tyrA* Δ*trpC* cheater strain (labeled "C") both in mono culture in YNB + glucose, either with no supplements or supplemented with tyrosine and tryptophan, and in co culture in YNB + glucose with either *S. cerevisiae* Δ*trp3* or *E. coli* Δ*tyrA* (labeled "P").

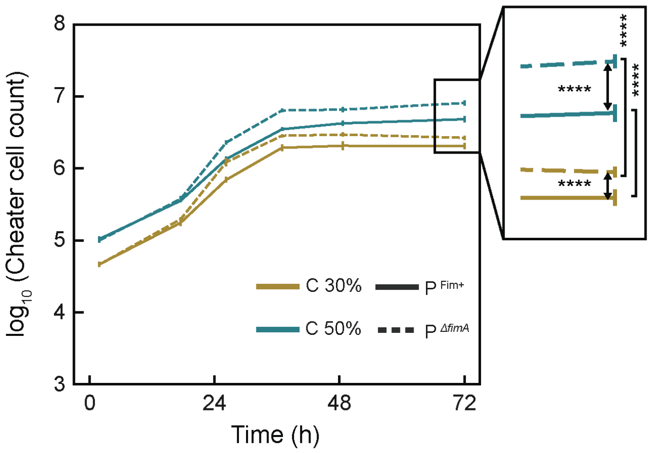

Figure S13. Cell count of cheater *E. coli* in the cross-feeding community

Number of cheater (labeled "C") *E. coli* cells measured by flow cytometry in the cross-feeding co-cultures with yeast and an *E. coli* partner (labeled "P") that is either fimbriated (straight lines) or fimbrialess (dotted line) as in figure 4C. Error bars represent standard deviations of three biological replicates. ****$p \leq 0.0001$ from a two-tailed *t*-test assuming equal variances of the data sets.

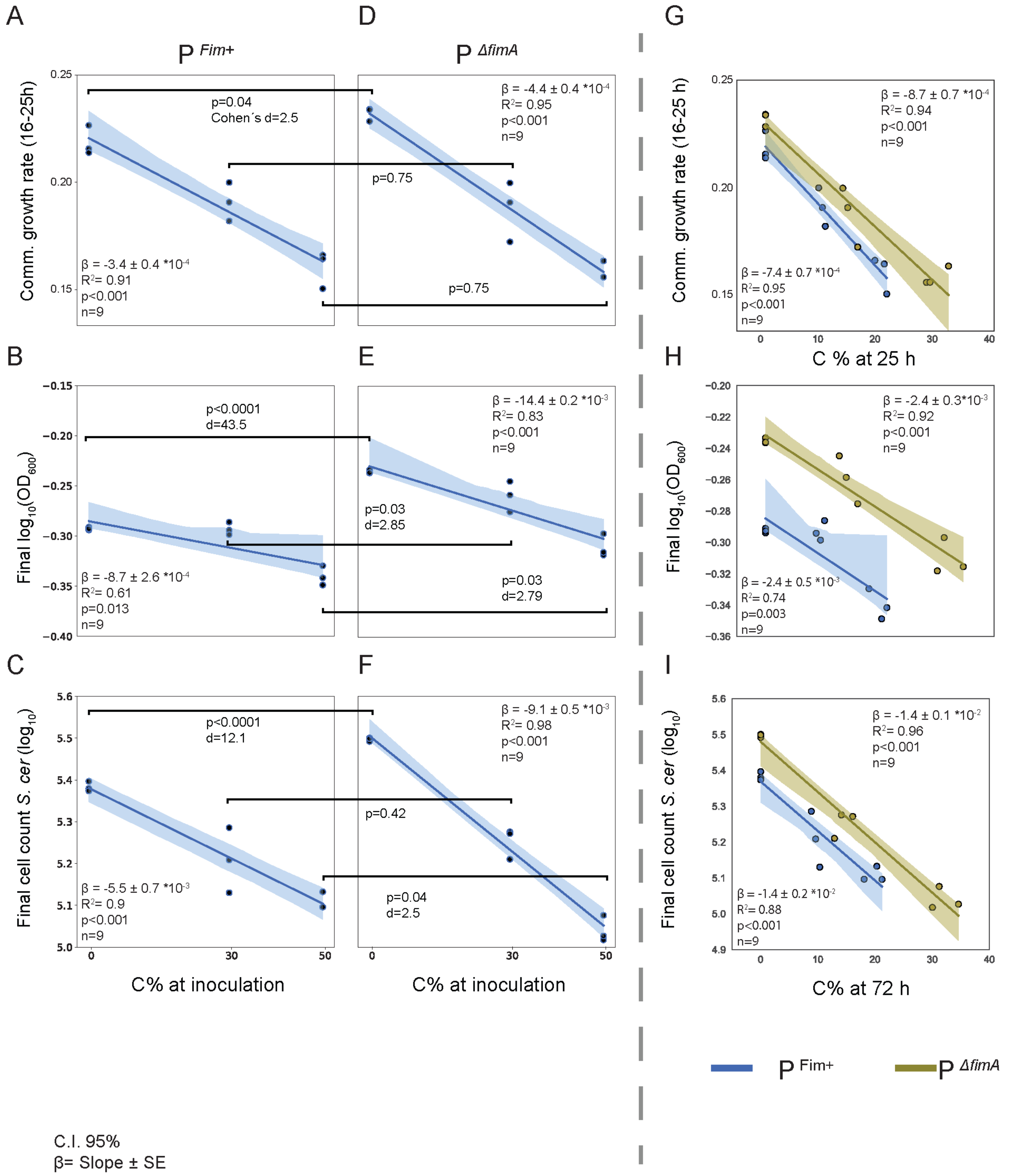

Figure S14. Dependence of the composition and growth of communities on the cheater fraction

A-F Dependence of the growth rate in exponential phase (A,D), of the total final OD600 (B,E) and of the final yeast cell count (C,F) from cocultures as in Figure 3C,E on the initial fraction of the cheater (labelled "C") at inoculation and either a fimbriated (left) or fimbrialess (right) *E. coli* partner (labelled "P"). **G-I** Same data but plotted against the final cheater fraction at the time of the measure, 25 h in (G) and 72 h in (H) and (I). Linear regression analysis and two-tailed *t*-test assuming equal variances of the data sets were performed. Each condition was assessed for three biological replicates, indicated as dots while shadings indicate a confidence interval of 95%. Regression line slopes, indicated as β, have been included along with the standard error.

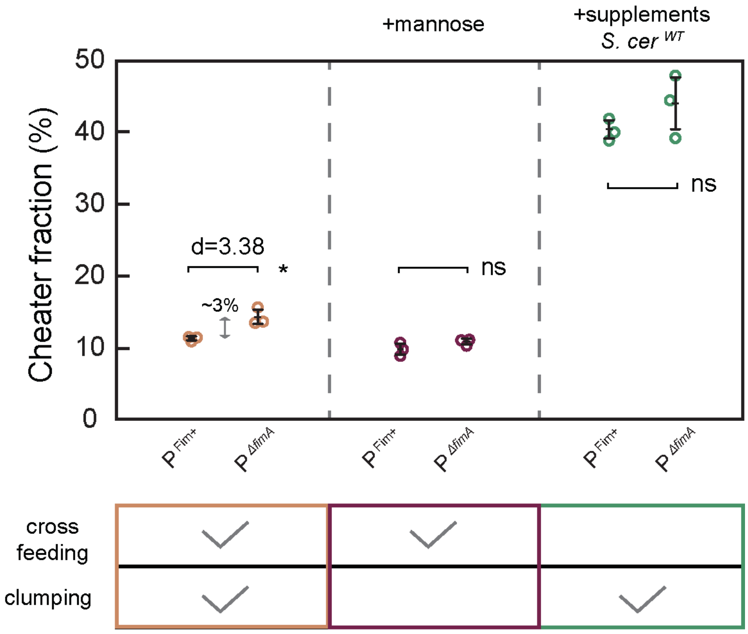

Figure S15. Effects of direct physical association between partners in presence of fimbrialess cheater

Fraction of a fimbrialess cheater (Δ*fimA*) in communities containing either Fim$^+$ or Δ*fimA E. coli* partner at the initial 50% abundance of cheater, grown in YNB-glucose (orange), YNB-glucose supplemented with 4% mannose (red) and in in YNB-glucose supplemented with CSM and with *S. cerevisiae* prototroph (green). **p* ≤0.05, *ns*=not significant in a two tailed *t*-test assuming equal variances of the data sets for three biological replicates, represented as circles.

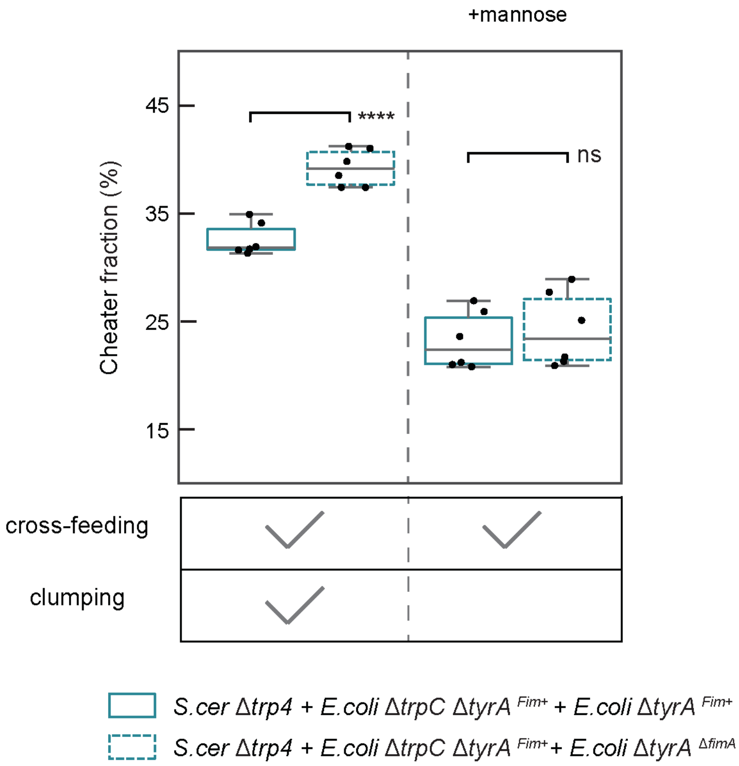

Figure S16. Protective effects of fimbriation against cheater in community with Δ*trp4* yeast strain

Fraction of fimbriated cheater in communities containing either Fim^+ (solid line boxes) or Δ*fimA* (dashed line boxes) *E. coli* partner at the initial 50% abundance of the cheater, grown either in YNB-glucose or YNB-glucose supplemented with 4% mannose, as indicated. *****p* ≤0.0001, *ns*=not significant in a two tailed *t*-test assuming equal variances of the data sets for six biological replicates represented as dots.

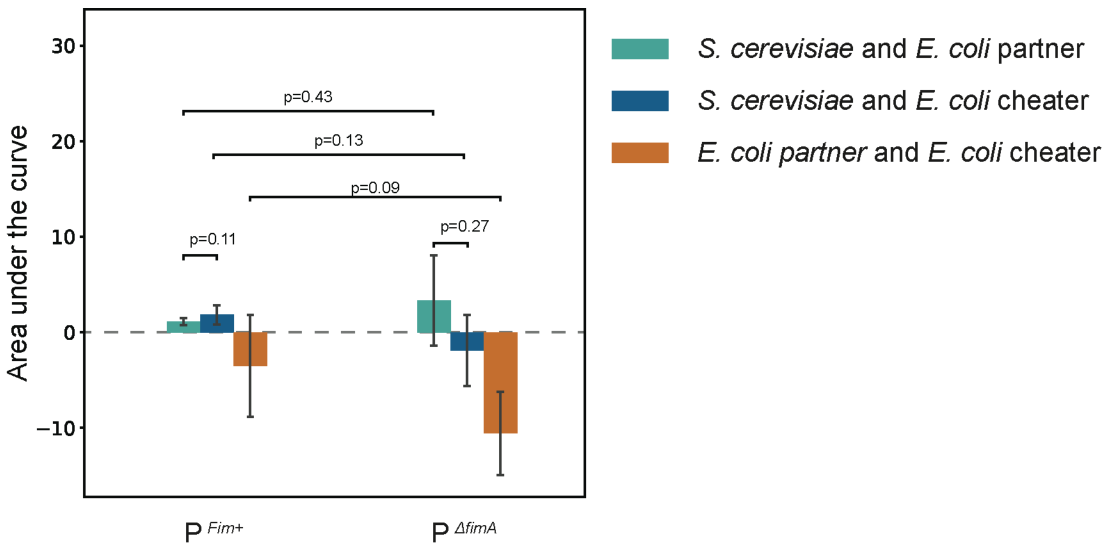

Figure S17. Cross-correlation between spatial arrangement of community members in presence of cheater strain

Area under the curve from cross correlations analysis (see Fig. S1) between different community members in sessile communities grown as in Figure 3G. Two-sided *t*-test assuming equal variance between data sets were performed. Each data set included four biological replicates.

A B

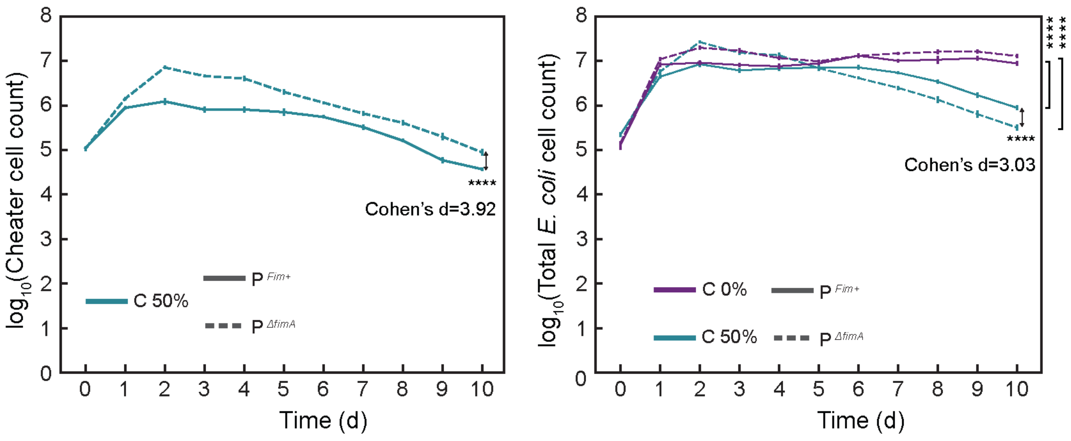

Figure S18. Cell count of cheater and of total *E. coli* population in semi-continuous co-culture

A,B Numbers of cheater (A) and total (B) *E. coli* cells measured by flow cytometry in in 20 µL of the same cross-feeding semi-continuous co-cultures as in Figure 4B. Error bars represent standard deviations of six to twelve biological replicates. *****p* ≤0.0001 in a two tailed *t*-test assuming equal variances of the data sets. Cohen´s *d* was calculated to quantify the effect size.

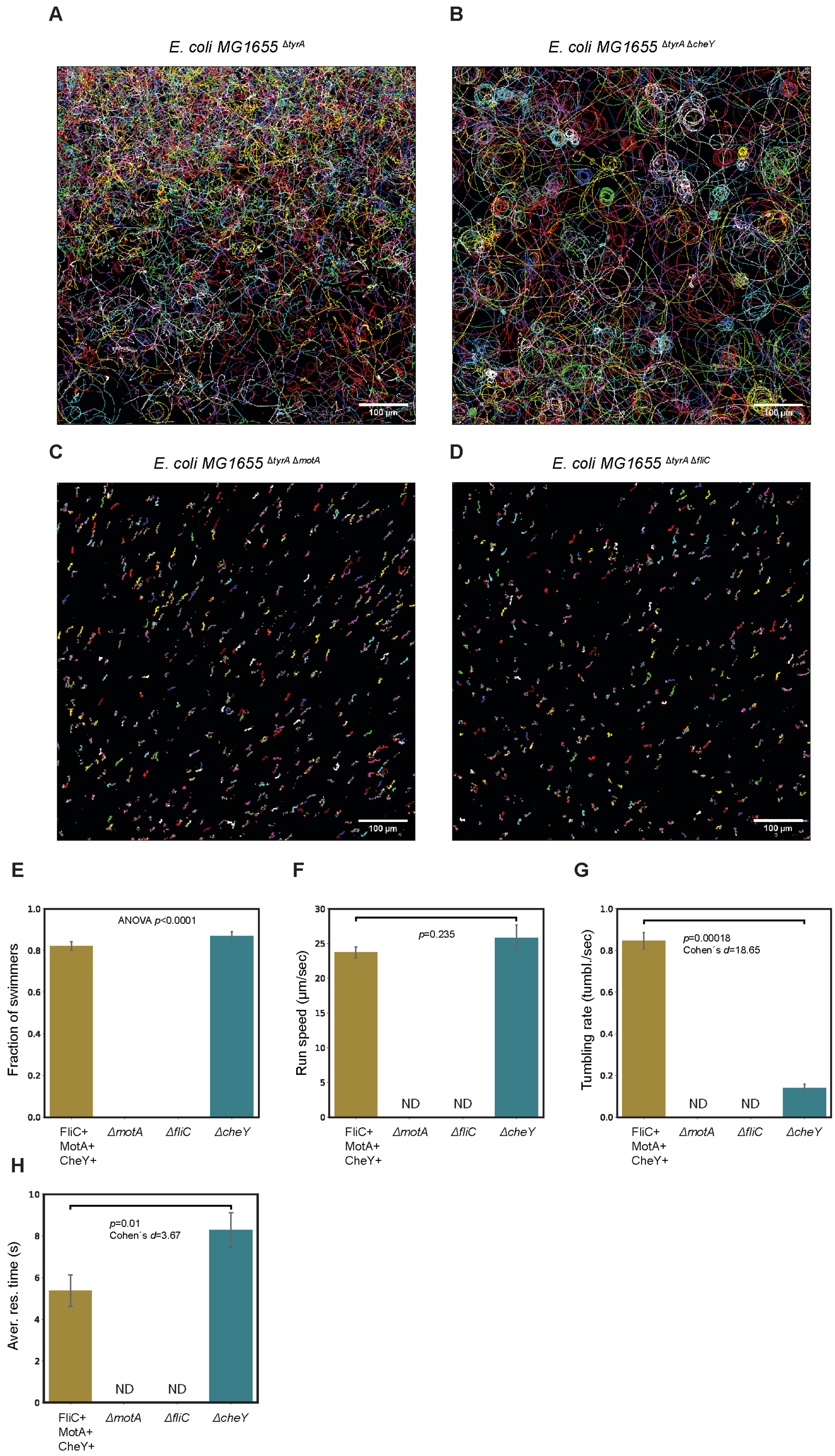

Figure S19. Characterization of motility phenotypes

A-D Particle tracking of *E. coli* strains used to assess influence of motility, for motility wildtype (A), Δ*cheY* (B), Δ*motA* (C), and Δ*fliC* (D). Each color represents the trajectory of a single bacterium. **E-H** Quantification of fraction

of swimmers (E), swimming speed (F), tumbling rate (G) and average residence time at the surface (H) for each strain. Of note, rare reorientation events in Δ*cheY* strain that are detected as tumbling are rather caused by cell collisions with other cells, surface defects or alike. Error bars represent the standard deviations of three biological replicates, each measuring at least fifty cell trajectories. One-way ANOVA and two-sided *t*-test assuming equal variance between data sets were performed.

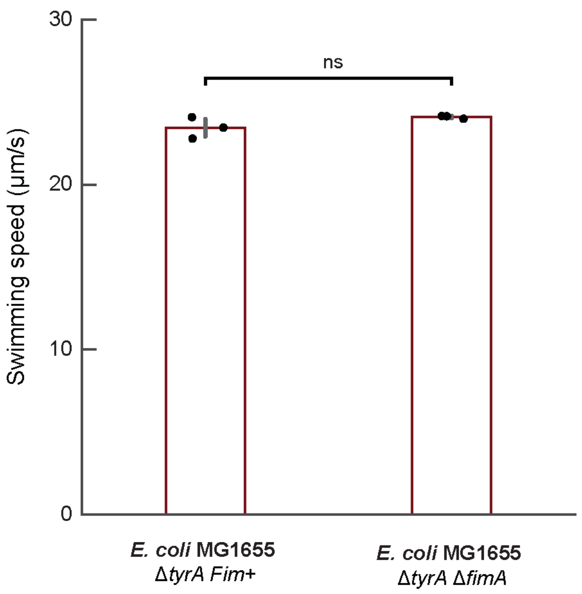

Figure S20. Fimbriation has no impact of motility

Swimming speed, measured as in Fig. S19, of fimbriated or fimbrialess *E. coli* cells grown in YNB fructose supplemented with CSM. *ns* from a *t*-test assuming equal variances between the samples for three biological replicates, indicated as dots, each measuring at least fifty cell trajectories.

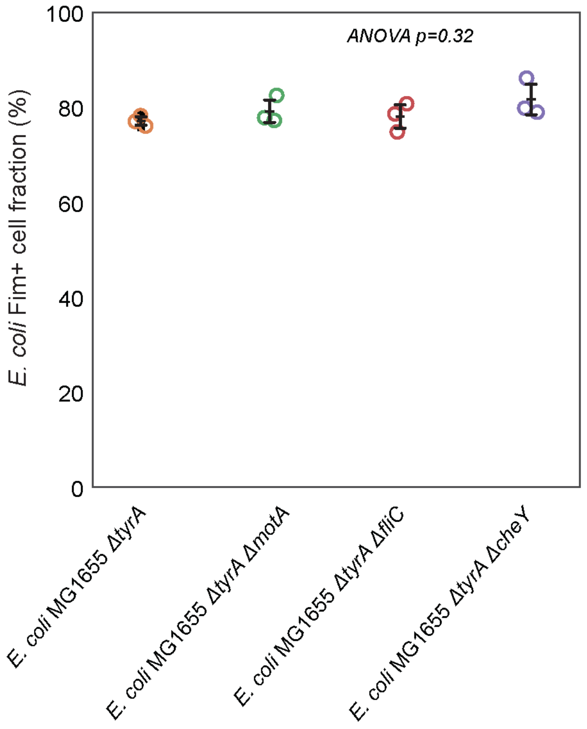

Figure S21. Activation status of the *fim* operon does not depend on motility

Quantification of the *fim* status of all the *E. coli* MG1655 partners used in this study, as described in Fig. S5, including the non-motile and non-chemotactic. *p* value from a one-way ANOVA test with three biological replicates per each strain indicated as circles.

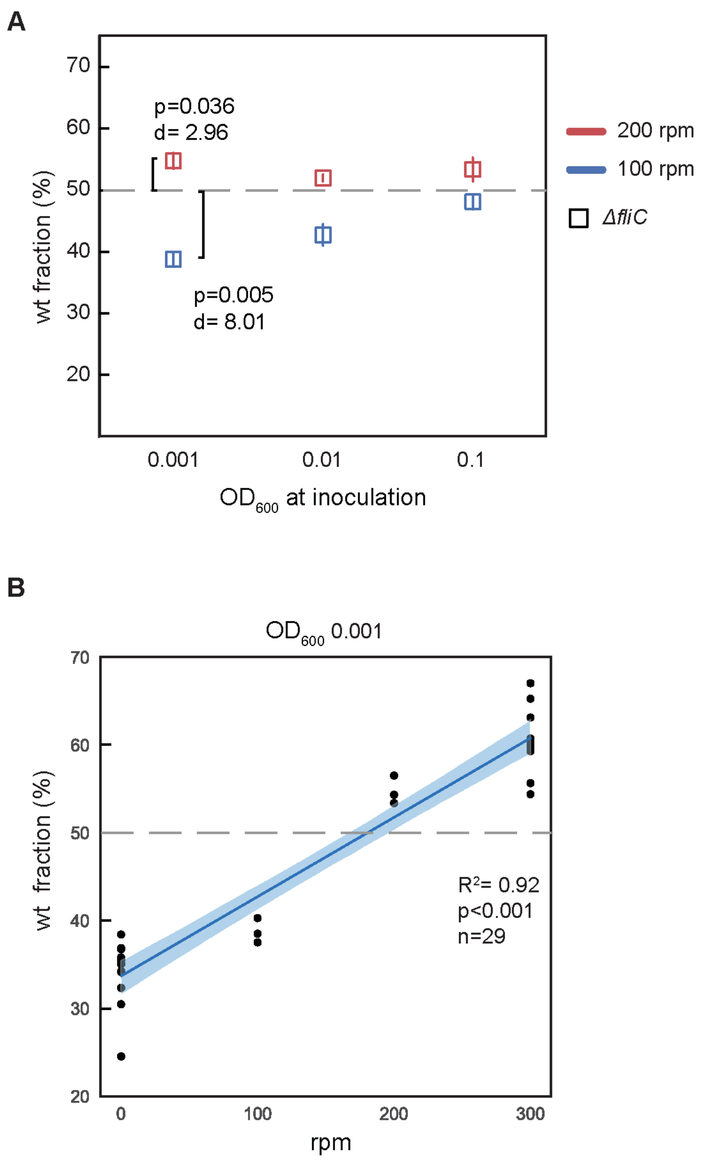

Figure S22. Dependence of fitness of motile cells on shaking rate in physically interacting community

A Fraction of motile *E. coli* cells (labeled with mCherry) compared to the total *E. coli* population in co-culture with sfGFP-labeled non-motile (Δ*fliC*) *E. coli* cells and with yeast at different shaking rates, as indicated. Communities were inoculated with different initial optical density (OD) as indicated and grown for 96 hours in YNB-glucose minimal medium. Error bars represent standard deviations of three biological replicates. One sample t- was performed. Cohen´s *d* was calculated to quantify the effect size. **B** Correlation analysis between motile strain cell fractions in FliC+ and Δ*fliC* co-cultures inoculated with an initial OD600 of 0.001 and the different shaking rates at which they were cultured. The linear regression analysis was performed with a sample size of 29 biological replicates.

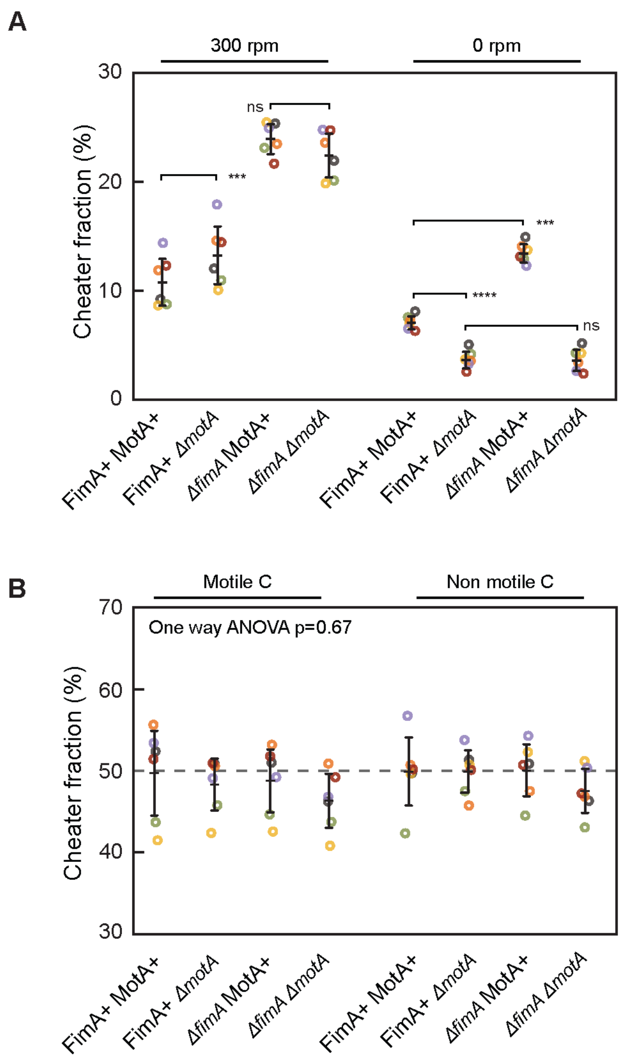

Figure S23. Effects of motility on communities containing cheater with or without cross feeding

A Cheater fraction within the total *E. coli* population in cross-feeding communities containing a non-motile and fimbriated *E. coli* cheater (labeled with sfGFP) co-cultured with yeast and with an mCherry-labeled *E. coli* partner displaying different status of fimbriation and motility, as indicated. Communities were inoculated with an initial 50% cheater fraction and an initial optical density of 0.001, and grown for 96 hours under shaking (300 r.p.m) or without shaking (0 r.p.m) in YNB-fructose minimal medium. Error bars are standard deviations of six biological replicates represented as circles. ****p<0.0001, ***p<0.001, **p<0.01 from paired t-test. **B** Cheater fraction in communities in absence of cross feeding, having either motile or non-motile fimbriated cheater strains (labeled "C"), in combination with indicated *E. coli* partner cells, inoculated with a total initial OD of 0.001 and grown in YNB fructose supplemented with CSM under shaking (300 r.p.m). p value from a one-way ANOVA test.

Table S1. Strains and plasmid used in this study

**Strains used in this study**		
Escherichia coli BW25113	[4]	
Escherichia coli MG1655	[5]	
Escherichia coli BW25113 Δ*tyrA*::kan^R	[6]	JW2581
Escherichia coli BW25113 Δ*trpC*::kan^R	[6]	JW1254
Escherichia coli BW25113 Δ*fliC*::kan^R	[6]	JW1908
Escherichia coli BW25113 Δ*cheY*::kan^R	[6]	JW1871
Escherichia coli BW25113 Δ*fimA*::kan^R	[6]	JW4277
Escherichia coli BW25113 Δ*trpC*::FRT Δ*tyrA*::kan^R	This manuscript	
Escherichia coli BW25113 Δ*fimA*::FRT Δ*tyrA*::kan^R	This manuscript	
Escherichia coli BW25113 Δ*fimA*::FRT Δ*trpC*::FRT Δ*tyrA*::kan^R	This manuscript	
Escherichia coli MG1655 Δ*motA*::FRT	[7]	
Escherichia coli MG1655 Δ*tyrA*::kan^R	This manuscript	
Escherichia coli MG1655 Δ*cheY*::FRT Δ*tyrA*::kan^R	This manuscript	
Escherichia coli MG1655 Δ*fliC*::FRT Δ*tyrA*::kan^R	This manuscript	
Escherichia coli MG1655 Δ*fimA*::FRT Δ*tyrA*::kan^R	This manuscript	
Escherichia coli MG1655 Δ*motA*::FRT Δ*tyrA*::kan^R	This manuscript	
Escherichia coli MG1655 Δ*fimA*::FRT Δ*fliC*::FRT Δ*tyrA*::kan^R	This manuscript	
Escherichia coli MG1655 Δ*fimAA*::FRT Δ*motA*::FRT Δ*tyrA*::kan^R	This manuscript	
Escherichia coli MG1655 Δ*trpC*::FRT Δ*tyrA*::kan^R	This manuscript	
Escherichia coli MG1655 Δ*motA*::FRT Δ*trpC*::FRT Δ*tyrA*::kan^R	This manuscript	
S. cerevisiae BY4741 (MATa his3Δ1 leu2Δ0 met15Δ0 ura3Δ0)	[8]	
S. cerevisiae BY4741 *Δtrp3*::*kanMX*^{R}	[9]	YKL211C
S. cerevisiae BY4741 *Δtrp4*::*kanMX*^{R}	[9]	YDR354W
S. cerevisiae BY4741 *Δhis3::HIS3-Pglk1-mTurquoise2-Tglk1*	This manuscript	
S. cerevisiae BY4741 *Δhis3::HIS3-Pglk1-mTurquoise2-Tglk1 Δtrp3*::*kanMX*^{R}	This manuscript	
S. cerevisiae BY4741 *Δhis3::HIS3-Pglk1-mTurquoise2-Tglk1 Δtrp4*::*kanMX*^{R}	This manuscript	
**Plasmids**		
pOB2 (pTrc99a::mCherry)	[10]	
pNB1 (pTrc99a::GFP)	[11]	
pISJ8	[12]	
pGS5 (*HIS3-Pglk1-mTurquoise2-Tglk1*)	This manuscript	

Table S2. Two-way ANOVA analysis

Table representing the results from a two-way ANOVA analysis followed by a Tukey HSD *post hoc* analysis for data presented in Fig. 2F.

ANOVA table	sum_sq	df	F	*p*
**Condition**	1576.00	2.00	318.70	9E-15
**Sample**	218.56	1.00	88.39	2E-08
**Interaction**	130.45	2.00	26.38	4E-06
**Residual**	44.51	18.00		

**Sample:**	**Condition:**
**fim+**	**CF** (crossfeeding)
**fim-**	**Mannose** (4% mannose)
	**Suppl** (+CSM compl.)

Tukey HSD *post hoc*

Condition

	group1	group2	Diff	Lower	Upper	q-value	*p*-value
**0**	mannose	Suppl	28.06	26.06	30.07	50.48	0.001
**1**	mannose	CF	11.28	9.27	13.28	20.28	0.001
**2**	Suppl	CF	16.79	14.47	19.11	26.15	0.001

Sample

	group1	group2	Diff	Lower	Upper	q-value	p-value
**0**	fim+	fim-	4.00	2.65	5.35	8.82	0.001

Interaction

	group1	group2	Diff	Lower	Upper	q-value	p-value
**0**	('mannose', 'fim+')	('mannose', 'fim-')	1.16	-1.73	4.04	1.80	0.772
**1**	('mannose', 'fim+')	('Suppl', 'fim+')	27.83	24.30	31.36	35.40	0.001
**2**	('mannose', 'fim+')	('Suppl', 'fim-')	29.45	25.92	32.99	37.46	0.001
**3**	('mannose', 'fim+')	('CF', 'fim+')	5.82	2.28	9.35	7.40	0.001
**4**	('mannose', 'fim+')	('CF', 'fim-')	17.89	14.36	21.42	22.75	0.001
**5**	('mannose', 'fim-')	('Suppl', 'fim+')	26.68	23.14	30.21	33.93	0.001
**6**	('mannose', 'fim-')	('Suppl', 'fim-')	28.30	24.77	31.83	35.99	0.001
**7**	('mannose', 'fim-')	('CF', 'fim+')	4.66	1.13	8.20	5.93	0.006
**8**	('mannose', 'fim-')	('CF', 'fim-')	16.73	13.20	20.27	21.28	0.001
**9**	('Suppl', 'fim+')	('Suppl', 'fim-')	1.62	-2.46	5.70	1.79	0.776
**10**	('Suppl', 'fim+')	('CF', 'fim+')	22.01	17.93	26.09	24.25	0.001
**11**	('Suppl', 'fim+')	('CF', 'fim-')	9.94	5.86	14.02	10.95	0.001
**12**	('Suppl', 'fim-')	('CF', 'fim+')	23.64	19.56	27.72	26.04	0.001

References

1. Zhang H, Susanto TT, Wan Y, Chen SL. Comprehensive mutagenesis of the fimS promoter regulatory switch reveals novel regulation of type 1 pili in uropathogenic *Escherichia coli*. *Proc Natl Acad Sci USA* 2016; **113**: 4182–4187.

2. Schindelin J, Arganda-Carreras I, Frise E, Kaynig V, Longair M, Pietzsch T, et al. Fiji: an open-source platform for biological-image analysis. *Nat Methods* 2012; **9**: 676–682.

3. Suchanek VM, Esteban-López M, Colin R, Besharova O, Fritz K, Sourjik V. Chemotaxis and cyclic-di-GMP signalling control surface attachment of *Escherichia coli*. *Mol Microbiol* 2020; **113**: 728–739.

4. Datsenko KA, Wanner BL. One-step inactivation of chromosomal genes in *Escherichia coli* K-12 using PCR products. *Proc Natl Acad Sci USA* 2000; **97**: 6640–6645.

5. Blattner FR, Plunkett G, Bloch CA, Perna NT, Burland V, Riley M, et al. The Complete Genome Sequence of *Escherichia coli* K-12. *Science* 1997; **277**: 1453–1462.

6. Baba T, Ara T, Hasegawa M, Takai Y, Okumura Y, Baba M, et al. Construction of *Escherichia coli* K-12 in-frame, single-gene knockout mutants: the Keio collection. *Mol Syst Biol* 2006; **2**: 2006.0008.

7. Laganenka L, López ME, Colin R, Sourjik V. Flagellum-mediated mechanosensing and RflP control motility state of pathogenic *Escherichia coli*. *MBio* 2020; **11**: e02269-19.

8. Winston F, Dollard C, Ricupero-Hovasse SL. Construction of a set of convenient *Saccharomyces cerevisiae* strains that are isogenic to S288C. *Yeast* 1995; **11**: 53–55.

9. Giaever G, Chu AM, Ni L, Connelly C, Riles L, Véronneau S, et al. Functional profiling of the *Saccharomyces cerevisiae* genome. *Nature* 2002; **418**: 387–391.

10. Amann E, Ochs B, Abel KJ. Tightly regulated tac promoter vectors useful for the expression of unfused and fused proteins in *Escherichia coli*. *Gene* 1988; **69**: 301–315.

11. Bellotto N, Agudo-Canalejo J, Colin R, Golestanian R, Malengo G, Sourjik V. Dependence of diffusion in *Escherichia coli* cytoplasm on protein size, environmental conditions and cell growth. *eLife* 2022; **11**: e82654.

12. Jensen SI, Lennen RM, Herrgård MJ, Nielsen AT. Seven gene deletions in seven days: Fast generation of *Escherichia coli* strains tolerant to acetate and osmotic stress. *Sci Rep* 2016; **5**: 17874.
